# Long non‐coding RNA LncTUG1 regulates favourable compression force‐induced cementocytes mineralization via PU.1/TLR4/SphK1 signalling

**DOI:** 10.1111/cpr.13604

**Published:** 2024-02-06

**Authors:** Han Wang, Tiancheng Li, Yukun Jiang, Shuo Chen, Zuping Wu, Xinyi Zeng, Kuan Yang, Peipei Duan, Shujuan Zou

**Affiliations:** ^1^ State Key Laboratory of Oral Diseases & National Center for Stomatology & National Clinical Research Center for Oral Diseases & Department of Orthodontics, West China Hospital of Stomatology Sichuan University Chengdu China; ^2^ Department of Orthodontics, Shanghai Ninth People's Hospital Shanghai Jiao Tong University School of Medicine, College of Stomatology, Shanghai Jiao Tong University, National Center for Stomatology, National Clinical Research Center for Oral Diseases, Shanghai Key Laboratory of Stomatology Shanghai China; ^3^ Stomatology Hospital, School of Stomatology, Zhejiang University School of Medicine, Zhejiang Provincial Clinical Research Center for Oral Diseases, Key Laboratory of Oral Biomedical Research of Zhejiang Province Cancer Center of Zhejiang University Hangzhou China

## Abstract

Orthodontic tooth movement (OTM) is a highly coordinated biomechanical response to orthodontic forces with active remodelling of alveolar bone but minor root resorption. Such antiresorptive properties of root relate to cementocyte mineralization, the mechanisms of which remain largely unknown. This study used the microarray analysis to explore long non‐coding ribonucleic acids involved in stress‐induced cementocyte mineralization. Gain‐ and loss‐of‐function experiments, including Alkaline phosphatase (ALP) activity and Alizarin Red S staining, quantitative real‐time polymerase chain reaction (qRT‐PCR), Western blot, and immunofluorescence analyses of mineralization‐associated factors, were conducted to verify long non‐coding ribonucleic acids taurine‐upregulated gene 1 (LncTUG1) regulation in stress‐induced cementocyte mineralization, via targeting the Toll‐like receptor 4 (TLR4)/SphK1 axis. The luciferase reporter assays, chromatin immunoprecipitation assays, RNA pull‐down, RNA immunoprecipitation, and co‐localization assays were performed to elucidate the interactions between LncTUG1, PU.1, and TLR4. Our findings indicated that LncTUG1 overexpression attenuated stress‐induced cementocyte mineralization, while blocking the TLR4/SphK1 axis reversed the inhibitory effect of LncTUG1 on stress‐induced cementocyte mineralization. The in vivo findings also confirmed the involvement of TLR4/SphK1 signalling in cementocyte mineralization during OTM. Mechanistically, LncTUG1 bound with PU.1 subsequently enhanced TLR4 promotor activity and thus transcriptionally elevated the expression of TLR4. In conclusion, our data revealed a critical role of LncTUG1 in regulating stress‐induced cementocyte mineralization via PU.1/TLR4/SphK1 signalling, which might provide further insights for developing novel therapeutic strategies that could protect roots from resorption during OTM.

## Graphical abstract

The schematic summary of proposed mechanisms for stress‐induced cementocytes mineralization. Under desirable compression stress, the expression of LncTUG1 in cementocytes was downregulated, which subsequently inhibited the direct interaction between PU.1 and promotor region of TLR4 and thus reduced the activity of TLR4/SphK1 signalling pathway. Then, the mineralization‐associated factors were upregulated and cementocytes mineralization was promoted.

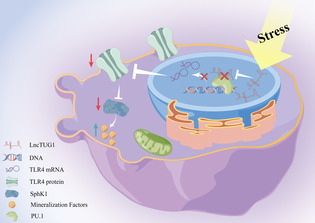

## 1 INTRODUCTION

Orthodontic tooth movement (OTM) is a common clinical practice for patients with malaligned teeth.^1^ During this treatment, favourable orthodontic forces initiate stress/strain distribution in the periodontal tissues, triggering signalling cascades that give rise to alveolar bone absorption and allow tooth movement but hardly induce external root resorption.^2^ However, the mechanisms of such antiresorptive properties of cementum under favourable orthodontic forces remain to be elucidated. Cementum is a mineralized connective tissue mainly composed of cementocytes, the mineralization of which functions as a barrier against root resorption.^3^, ^4^ Our preliminary study indicated that cementocytes could respond to mechanical stresses with subsequent changes in mineralization properties.^4^, ^5^ Thus, further investigation into the underlying mechanisms of mechanical stimuli on cementocyte mineralization is vital for understanding the antiresorptive properties of cementum during OTM.

Long non‐coding ribonucleic acids (lncRNAs) are a class of non‐coding RNAs with lengths typically exceeding 200 nucleotides.^6^ Many studies have shown that lncRNAs diversely participate in various cellular biological processes, such as embryonic development and cell differentiation.^7^, ^8^, ^9^, ^10^ Specifically in the skeletal system, lncRNAs are extensively involved in osteogenic mineralization by modulating the expression of mineralization genes at the transcriptional or post‐transcriptional levels.^10^, ^11^, ^12^ For instance, lncRNA SNHG14 could stabilize NEDD4L at the transcriptional level and further promote FOXA2 ubiquitination, enhancing the osteogenic mineralization of bone marrow‐derived mesenchymal stem cells.^13^ In a post‐transcriptional regulation manner, lncRNA SNHG1 upregulated DNA methyltransferase 1 (DNMT1) expression, resulting in osteoprotegerin (OPG) hypermethylation and decreased OPG expression, in turn contributing to osteoporosis.^14^ Recent studies have demonstrated that lncRNAs mediate mechanical stress‐induced osteogenic mineralization of periodontal ligament stem cells (PDLSCs) to control periodontal homeostasis.^15^, ^16^ Also playing a role in periodontal homeostasis and acting as the primary root cell type to sense orthodontic forces,^17^ cementocytes could likewise transduce mechanical signals^5^, ^17^ into biological responses that modulate cementum mineralization in a lncRNAs‐based regulatory manner. However, the potential mechanisms of such cellular events are yet to be studied.

Toll‐like receptors (TLRs) are type I transmembrane glycoproteins widely expressed in most mammalian cells and initiate the primary immune response and modulate other cellular functions such as osteogenesis.^18^, ^19^ Toll‐like receptor 4 (TLR4) is one of the most representative TLRs involved in regulating bone metabolism.^20^, ^21^, ^22^ Several lines of evidence revealed that TLR4 played an inhibitory role in bone formation by blocking the canonical Wnt/β‐catenin pathway.^20^, ^21^ The regulatory effect of TLR4 on osteogenesis was also observed in periodontal tissues; suppression of TLR4 could partially restore the osteogenic differentiation of PDLSCs, promoting periodontal regeneration.^23^, ^24^ Mechanistically, lncRNAs would very likely become the upstream regulators of TLR4 during osteogenic mineralization in periodontium, for instance, acting as miRNA sponges to modulate TLR4‐mediated osteogenic differentiation of PDLSCs.^25^ However, the regulatory effects of TLR4 on the mineralization of cementocytes, as another important cellular component of periodontal tissues, and whether such mechanisms are associated with lncRNAs have rarely been reported.

We determined and investigated the roles of lncRNA taurine‐upregulated gene 1 (LncTUG1), a novel mineralization regulator of cementocytes in stress‐induced cementum mineralization. LncTUG1 decreased in force‐loaded cementocytes and negatively regulated stress‐induced cementocyte mineralization by activating the TLR4/SphK1 axis. We also performed in vivo experiments to confirm the critical role of TLR4/SphK1 signalling in orthodontic force‐induced cementocyte mineralization during OTM. Mechanistically, we showed that LncTUG1 could directly bind and recruit PU.1 protein. This transcription factor (TF) recognized the promoter region of TLR4 and thus regulated the downstream signalling that targeted mineralization‐associated markers of cementocytes under favourable mechanical forces. Overall, these findings unveiled the LncTUG1‐based molecular mechanism of stress‐induced cementocyte mineralization, suggesting that the TLR4/SphK1 axis might serve as a promising therapeutic target to promote the antiresorptive activity of root cementum during OTM.

## 2 MATERIALS AND METHODS

### 2.1 Cell culture and mechanical loading

The immortalized murine cementocyte cell line IDG‐CM6 was created as previously described.^4^ Briefly, IDG‐CM6 cells were proliferated with 50 U/mL IFN‐γ (Interferon γ, Gibco, Grand Island, NY, USA) at 33°C and after reaching 80% confluence in 2 days, monolayer cells were cultured with differentiation media containing 50 mg/mL ascorbic acid and 4 mM β‐glycerophosphate, without INF‐γ, and incubated at 37°C. After 21 days, the cells were prepared for mechanical loading.

Static compression force was applied to IDG‐CM6 cells as described previously.^26^ Briefly, a glass cylinder was placed over the cell layer and the compression force was adjusted by adding stainless steel beads into the cylinder. The magnitude of compression force was set as 0.5 g/cm^2^ (desirable force) according to a previous study.^27^ Cells cultured in the absence of force were served as the control group. Cells were harvested at 6 h after the application of compression force for further experiments.

### 2.2 Adenovirus infection, plasmid construction, and stable transfection

The LncTUG1 overexpression adenoviruses and TLR4, SphK1, PU.1, FOXA2 overexpression plasmids as well as the siRNAs targeting LncTUG1, TLR4, SphK1, PU.1, FOXA2, and the luciferase vectors containing binding sequences of PU.1 with LncTUG1 or empty vector were all synthesized by Hanheng Biotechnology Co., Ltd., Shanghai, China. The constructs were all confirmed by sequencing. Afterwards, IDG‐CM6 cells were seeded at 30%–50% confluence and infected with a LncTUG1 overexpression adenovirus (at an Multiplicity of infection of 45), a TLR4 or a SphK1 or a PU.1 or a FOXA2 overexpression plasmid and si‐LncTUG1, si‐TLR4, si‐SphK1, si‐PU.1, and si‐FOXA2 using a conventional transfection reagent (Lipofectamine 2000, Invitrogen, Waltham, MA, USA). After 48 h infection, the fresh medium containing puromycin was used to replace the previous medium and eliminate non‐infected cells. The transfection efficiency was determined by quantitative real‐time polymerase chain reaction (qRT‐PCR)  and Western blot. The same method was used to transfect the negative control adenoviruses and plasmids, which referred as NC in names of groups. Co‐transfection of plasmids was performed in the same methods and indicated as follows: NC + oe‐Lnc, NC + oe‐TLR4, NC + si‐TLR4, oe‐Lnc + si‐TLR4, NC + oe‐SphK1, oe‐TLR4 + si‐SphK1, NC + oe‐PU.1 and oe‐Lnc + si‐PU.1.

### 2.3 Quantitative real‐time PCR assays

Total RNA was extracted using Trizol reagent (Invitrogen, Carlsbad, CA, USA). After detecting the RNA concentration, samples were reversely transcribed to cDNA with SuperScript® III cDNA Synthesis Kit (Invitrogen, Carlsbad, CA, USA). The qRT‐PCR was conducted with the Quant Studio 3 Real‐Time PCR Systems (Thermo Fisher Scientific, San Jose, CA, USA) using TB Green™ Premix Ex Taq™ II (Takara, Tokyo, Japan). Gene expression level was normalized against glyceraldehyde‐3‐phosphate dehydrogenase (Gapdh). Relative gene expression was calculated using the arithmetic formula 2^−△△Ct^ method. The mRNA primer sequences used in this study were listed in Table S1.

### 2.4 Western blot assays

Western blotting was conducted as previously described.^17^ Briefly, the samples were loaded onto Sodium dodecyl sulphate‐polyacrylamide gel for electrophoresis and transferred to polyvinylidene difluoride membranes. Membranes were blocked with 5% BSA and incubated with primary antibodies at 4°C overnight. Primary antibodies were listed in Table S2. Anti‐GAPDH was used as an internal control. Proteins were detected using horseradish peroxidase‐conjugated secondary antibodies (ZSGB‐Bio, Beijing, China) at room temperature for 1 h and visualized using ClarityTM Western ECL Substrate (Bio‐Rad, Hercules, CA, USA). The intensity of each band was visualized with a ChemiDoc Touch Imaging System (Bio‐Rad, USA) and quantified after normalization to GAPDH with Image‐Pro Plus 6.0 (Media Cybernetics, Bethesda, MD, USA).

### 2.5 ALP activity and Alizarin Red S staining

Total protein content of supernatant of cell samples was measured by a protein assay kit (Beyotime, Shanghai, China). Then Alkaline phosphatase (ALP) enzyme activity was determined using a quantitative ALP assay kit (Beyotime, Shanghai, China) according to the manufacturer's instructions. For Alizarin red S (ARS) staining, cells were stained with 2% (wt/vol) ARS solution (pH 4.2, Sigma‐Aldrich, St. Louis, MO, USA) and observed under an Olympus IX70 microscope (Olympus, Tokyo, Japan). For quantitative calcium measurement, 10% cetylpyridinium chloride (J&K Chemical, Beijing, China) solution was then added to each well for elution of the dye and after shaking for 1 h, samples of the resulting solution were then loaded into a 96‐well plate and read at 570 nm.

### 2.6 RNA sequencing and data analysis

Total RNA was extracted from IDG‐CM6 cells under desirable compression force (0.5 g/cm,^2^ 6 h) and subjected to RNA‐sequencing analysis using the Illumina Hiseq 2500 platform (Novogene Bioinformatics Technology Co., Ltd., China). Transcriptome assembly was accomplished using Trinity software. The clean reads of each sample were mapped to the reference sequence using RSEM software. The transcripts were clustered by Corset software. Gene expression level was standardized using DESeq software. The individual genes were considered as differentially expressed genes (DEGs) when the adjusted *p* between two treatments was <0.05. DEG heat maps were clustered by k‐means clustering using the Euclidean distance as the distance and observed using Java TreeView software. Gene ontology (GO) analysis was performed with the DAVID online tool. Top GO categories were selected according to the *p*‐values. Kyoto Encyclopedia of Genes and Genomes (KEGG) is a database resource for understanding high level functions and utilities of the biological system (http://www.genome.jp/kegg/). We used KOBAS software (KOBAS, Surrey, UK) to examine the statistical enrichment of differential expression genes in KEGG pathways.

### 2.7 Immunofluorescence staining (cell sample)

For immunofluorescence assays, cells were then fixed for 15 min at room temperature with 4% Paraformaldehyde (PFA)  and washed two times with Phosphate buffer solution (PBS). After fixation, cells were penetrated with 0.5% Triton X‐100 (Beyotime, Shanghai, China) for 10 min, and blocked with 5% Bovine serum albumin (BSA) for 1 h. After being washed with PBS three times, primary antibodies (listed in Table S2) were added into the dishes and incubated at 4°C overnight, respectively. A secondary antibody conjugated to Cy3 (Beyotime, Shanghai, China) was used to incubate the samples for 2 h. 4′,6‐Diamidino‐2‐phenylindole (DAPI; Sigma, St. Louis, MO, USA) and phalloidine (6 μmol/L, Invitrogen, CA, USA) were applied to stain the nuclei and cytoskeleton. The cells were then observed with a confocal laser scanning microscopy (Olympus, Japan).

### 2.8 Luciferase reporter assays

Cells were seeded at a density of 5 × 10^4^ cells per well in a 24‐well plate. Cells were transfected with the reporter plasmid TLR4 or the corresponding negative control plasmid, the LncTUG1 overexpression adenovirus or the corresponding negative control adenovirus and the PU.1 (*Spi1*) overexpression plasmid or the corresponding negative control plasmid by using LipoFiter 3.0 according to the manufacturer's instructions (Hanheng Biotechnology Co., Ltd., Shanghai, China). The Renilla luciferase sequence in the pRL‐TK vector (Hanheng Biotechnology Co., Ltd., Shanghai, China) was used as an internal control. The luciferase activity was tested 48 h after transfection by using the Dual‐Luciferase Reporter Assay System (Promega, Beijing, China) and observed with a luminometer (Perkin Elmer). Relative luciferase activity was normalized to Renilla luciferase activity. All plasmids were synthesized by Hanheng Biotechnology Co., Ltd., Shanghai, China.

### 2.9 Subcellular RNA fractionations

Nuclear and cytoplasmic RNA fractionations were conducted using the PARIS™ Kit (Invitrogen, CA, USA) following the manufacturer's instruction, analysed by qRT‐PCR with *U6* small nuclear RNA and *Gapdh* mRNA as the nuclear and cytoplasmic endogenous control, respectively.

### 2.10 RNA fluorescence in situ hybridization and co‐localization assays

The cells were prepared for fluorescence in situ hybridization (FISH) using the methods previously described.^28^ After fixation with 4% formaldehyde containing 1/1000 diethyl pyrocarbonate at room temperature for 20 min, the cell layers were digested with 3% pepsin diluted in citrate buffer at 37°C for 2 min. The samples were incubated at 37°C in pre‐hybridization solution for 2–4 h and then hybridized with the FISH probes overnight. After hybridization, the samples were washed with wash buffer I (2 × SSC) for 5 min, wash buffer II (0.5 × SSC) for 15 min, wash buffer III (0.2 × SSC) for 15 min at 37°C. The samples were blocked at 37°C for 30 min and the Biotin‐Mouse Anti‐Digoxin was added for 120 min at 37°C after hybridization. SABC‐CY3 was used as the secondary antibody and then DAPI was stained for 10 min before samples were sealed with cover slips. For co‐localization studies, after RNA FISH, immunofluorescence staining was performed as described previously (2.7 Immunofluorescence staining). A secondary antibody conjugated to Cy5 (Beyotime, Shanghai, China) was used to incubate the samples for 2 h. All experiments were repeated three times. All FISH probes were commercially synthesized by Boster, Wuhan, China.

### 2.11 RNA immunoprecipitation

RNA immunoprecipitation (RIP) assay was performed using the Imprint RIP Kit (Sigma‐Aldrich, St. Louis, USA) with anti‐PU.1 Ab (Abcam, UK) or Immunoglobulin G (IgG) (Millipore) to examine the interaction between LncTUG1 and PU.1. The IDG‐CM6 cells were harvested and lysed using RIPA lysis buffer (Invitrogen, USA) containing magnetic bead‐bound anti‐PU.1 or the negative control IgG. The immunoprecipitated RNA was then isolated and the level of LncTUG1 was determined by qRT‐PCR and Gapdh was used as the endogenous control.

### 2.12 RNA pull‐down assay

RNA pull‐down assay was performed by the Bersin‐Bio™ RNA pulldown Kit (BersinBio, Guangzhou, China) according to the manufacturer's instructions. Briefly, LncTUG1 RNAs were transcribed and labelled by the Biotin in vitro and lacZ probe was used as the control. Using RNA structure buffer and RNase‐free water, the secondary structure of biotinylated LncTUG1 (probe) was established. Then, the probe was incubated with streptavidin‐coated magnetic beads for 2 h to generate probe‐bound magnetic beads. The cell lysates were incubated with the probe‐bound magnetic beads for 2 h and after separation, the protein level of PU.1 in the complex of RNA‐bound proteins was examined by western blotting.

### 2.13 Chromatin immunoprecipitation assays

Chromatin immunoprecipitation (ChIP) assays were conducted as described previously using the ChIP assay kit (Bes5001, BersinBio, Guangzhou, China) according to the manufacturer's instructions.^29^ The total of 2 × 10^7^ cells were washed with PBS and fixed with 1% formaldehyde to crosslink DNA at room temperature. After fragmenting the DNAs, the samples were enriched with antibodies and further inverse‐crosslinked at 65°C overnight. The qRT‐PCR analysis was performed to determine the amount of DNA pulled down. The IgG (Millipore) was used as negative control. The sequences of the primers used in the ChIP‐PCR assays were listed in Table S3.

### 2.14 Animals and application of orthodontic devices

Twenty 6‐week‐old male Wistar rats weighing 200 ± 10 g were used in this study. All experimental procedures were approved by the Ethics Committee of West China Hospital of Stomatology (WCHSIRB‐D‐2021‐207). The animals were anaesthetised with pentobarbital sodium (40 mg/kg body weight). An orthodontic closed coil spring (Grikin Advanced Materials, Beijing, China) was inserted between the maxillary left first molar (M1) and the incisor to achieve orthodontic molar movement with 0 g (Control group) and 25 g (Stress group). Stress was also combined with daily intraperitoneal injection of a TLR4‐specific agonist Lipopolysaccharide (LPS) (2 mg/kg, L2630, Millipore, Sigma‐Aldrich, St. Louis, MO, USA)^30^ or with intraperitoneal injection of an SphK1 activator (0.05 mg/kg, K6PC‐5, Selleck, Beijing, China),^31^, ^32^ which were divided as Stress + LPS group and Stress + K6PC‐5 group, respectively. After 14 days, all experimental rats were euthanized by an overdose of pentobarbital and alveolar bone blocks that included M1 were harvested for further analysis. Each group included five rats.

### 2.15 Microcomputed tomography analysis

All the samples were scanned using a high‐resolution microcomputed tomography (micro‐CT) 50 system (Scanco Medical, Brüttisellen, SUI) with a voxel resolution of 10 μm, passing through a three‐dimensional Gaussian filter (mean, 1.2; filter support, 1). Mimics 21.0 software (Materialize, Leuven, Belgium) was used to reconstruct the three‐dimensional model of the alveolar bone block. To determine the severity of root resorption, the distobuccal root of each sample was separated and the total volume of the resorption pits at mesial surface of the distobuccal root was calculated using the distobuccal roots of M1 on the contralateral side as the reference.^17^

### 2.16 Histological, immunohistochemical, and immunofluorescence staining (tissue sample)

After micro‐CT scanning, the specimens were fixed in 10% formalin for 24 h followed by decalcification in 14% EDTA (pH 7.1) and embedded in paraffin and were cut into 5‐μm sections. Selected sections were treated with haematoxylin and eosin (HE, Solarbio, Beijing, China) and Masson's trichrome (G1340, Solarbio, Beijing, China) staining according to the manufacturers' instructions. Each slide was sealed with resinene and viewed under the light microscope.

Immunofluorescence staining was performed to observe β‐catenin in cementocytes at apical third of root cementum. Tissue sections were heated at 95°C for antigen retrieval for 30 min, washed 3 times with PBS for 5 min each, and blocked in 10% goat serum for 30 min at 37°C. Then, the samples were incubated with rabbit anti‐β‐catenin (ET1601‐5, 1:1000, Huabio, Hangzhou, China) overnight at 4°C. Then, slides were incubated with the Alexa Fluor 594 donkey anti‐rabbit (ab150108, 1:200, Abcam, Shanghai, China) for 2 h at 37°C and counterstained with DAPI (Sigma, St. Louis, MO, USA) for 30 min. After three washes with PBS, stained sections were observed using fluorescent microscopy (DMI 6000; Leica, Wetzlar, Germany). Quantitative analyses of the images were done by the Image‐Pro Plus 6.0 Software (Media Cybernetics, Bethesda, MD, USA).

Immunohistochemical staining was performed to observe the expressions of Runx2 and Osterix in the cementocytes using primary antibody ET1612‐47 at a 1:300 dilution (Huabio, Hangzhou, China) and ER1914‐47 at a 1:300 dilution (Huabio, Hangzhou, China), respectively. The means of integrated optical density of the immunohistochemical staining were analysed by Image‐Pro Plus 6.0 Software (Media Cybernetics, Bethesda, MD, USA).

### 2.17 Statistical analysis

Statistical analysis was conducted using the SPSS 22.0 Software package (IBM Corporation, Armonk, NY, USA). Data were presented as line graph with mean values ± SD and boxplot with median value (cross), interquartile range (box), minimum/maximum (whiskers) and symbols representing all data points from at least five independent experiments. Statistical comparison was performed using two‐tailed Student's *t*‐test or one‐way analysis of variance with Tukey's post hoc test. A *p* < 0.05 was considered statistically significant.

## 3 RESULTS

### 3.1 Favourable compressive force promoted the mineralization of IDG‐CM6 cells

The qRT‐PCR and Western blot results revealed that the expressions of mineralization‐associated genes β‐catenin, ALP, Osterix, Runx2, and Col1a1 significantly increased under compression force (Figure S1A–C). Consistently, the ARS staining showed more mineralized nodule formation, and the activity of ALP was significantly elevated in the stress group (Figure S1D,E). These results demonstrated that favourable compressive force promoted the mineralization of IDG‐CM6 cells.

### 3.2 LncTUG1 negatively regulated the stress‐induced mineralization of IDG‐CM6 cells

**FIGURE 1:**
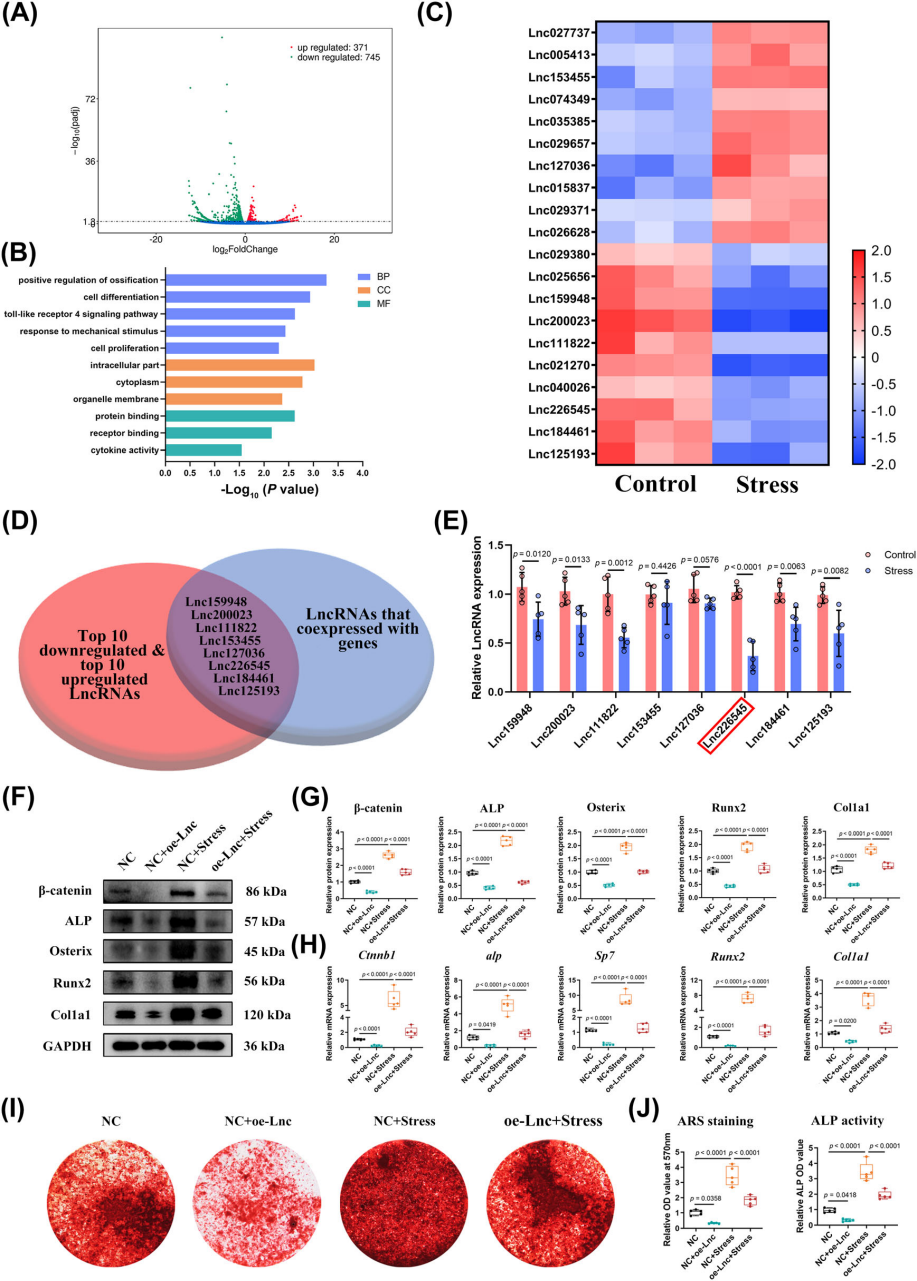
Long non‐coding ribonucleic acid (lncRNA) taurine‐upregulated gene 1 (LncTUG1) negatively regulates the stress‐induced mineralization of IDG‐CM6 cells. (A) The volcano plot displayed global gene expression in the IDG‐CM6 cells under desirable compression force. Green represents downregulated genes while red represents upregulated genes. (B) Gene ontology (GO) enrichment analysis of genes co‐expressed with differentially expressed lncRNAs (>1.5‐fold) in IDG‐CM6 cells under desirable compression force. (C) Heatmap of the top 10 downregulated and top 10 upregulated lncRNAs in the IDG‐CM6 cells under desirable compression force. Red represents upregulated lncRNAs while green represents downregulated lncRNAs under mechanical force. (D) The Venn diagram was performed to screen out the potential lncRNAs that involved in stress‐induced cementocytes mineralization. (E) Verification of the 8 selected lncRNAs using qRT‐PCR analysis and focusing on LncTUG1 for further experiments. (F,G) The expressions of β‐catenin, ALP, Osterix, Runx2, and Col1a1 in IDG‐CM6 cells, after overexpression of LncTUG1 and/or application of desirable compression force, were assessed by western blot analysis using GAPDH as a loading control and the corresponding quantitative analysis was performed. (H) The qRT‐PCR analysis was performed to examine the mRNA levels of *Ctnnb1*, *alp*, *Sp7*, *Runx2*, and *Col1a1* in IDG‐CM6 cells. (I,J) Representative images and quantitative analysis of Alizarin Red S (ARS) staining as well as the activity of ALP were conducted in IDG‐CM6 cells. Statistical comparison was performed using one‐way analysis of variance with Tukey's post hoc test. *p* < 0.05 was considered statistically significant. At least five independent experiments were conducted.

To further identify the potential lncRNAs involved in IDG‐CM6 cell mineralization, we performed RNA‐seq on IDG‐CM6 cells under a favourable compressive force for 6 h. Overall, 371 lncRNAs were significantly upregulated, and 745 were significantly downregulated in the favourable compressive force group compared with the control group (Figure 1A). The GO analysis showed that the genes co‐expressed with differentially expressed lncRNAs, indicating that those mineralization‐associated genes sharing similar trends with those differentially expressed lncRNAs were enriched for associations with positive regulation of ossification, cell differentiation, response to mechanical stimulus, and protein binding (Figure 1B). The heatmap in Figure 1C shows the symbols of the top 10 upregulated lncRNAs and the top 10 downregulated lncRNAs in the stress group. Taking the intersections of the 20 lncRNAs selected above and the differentially expressed lncRNAs that co‐expressed with genes, 8 lncRNAs, Lnc159948, Lnc200023, Lnc111822, Lnc153455, Lnc127036, Lnc226545, Lnc184461, and Lnc125193 were involved in verifying the microarray results (Figure 1D). Based on the qRT‐PCR results, six of the 8 lncRNAs (Lnc159948, Lnc200023, Lnc111822, Lnc226545, Lnc184461, and Lnc125193) showed the same trend as the microarray results predicted; that is, they significantly decreased in the stress group compared with the control group, whereas the other 2 lncRNAs (Lnc153455 and Lnc127036) were not significantly different between the two groups (Figure 1E). According to this finding, Lnc226545 (LncTUG1) was the most downregulated lncRNA (highlighted in the red box in Figure 1E) in the stress group compared with the control group. Thus, the biological functions and underlying molecular mechanisms of LncTUG1 in stress‐induced cementocyte mineralization were further explored in this study.

To verify the role of LncTUG1 in stress‐induced cementocyte mineralization, we used lentivirus to overexpress and siRNA to knock down the LncTUG1, the efficiency of which was verified by qRT‐PCR analysis (Figure S1F). The results indicated that the expressions of mineralization‐associated genes β‐catenin, ALP, Osterix, Runx2, and Col1a1 decreased significantly after the overexpression of LncTUG1, while it increased after the knockdown of LncTUG1, compared with the NC (negative control) group (Figure 1F–H and Figure S1G–I). Furthermore, the promotion effect of favourable compressive force on those mineralization‐associated genes was partly reversed by the overexpression of LncTUG1 (Figure 1F–H), while it was partly enhanced by the knockdown of LncTUG1 (Figure S1G,H). Similar trends of mineralized nodule formation and the activity of ALP are shown consistently in Figures 1I,J and S1I.

### 3.3 LncTUG1 positively correlated with the expression of TLR4

**FIGURE 2:**
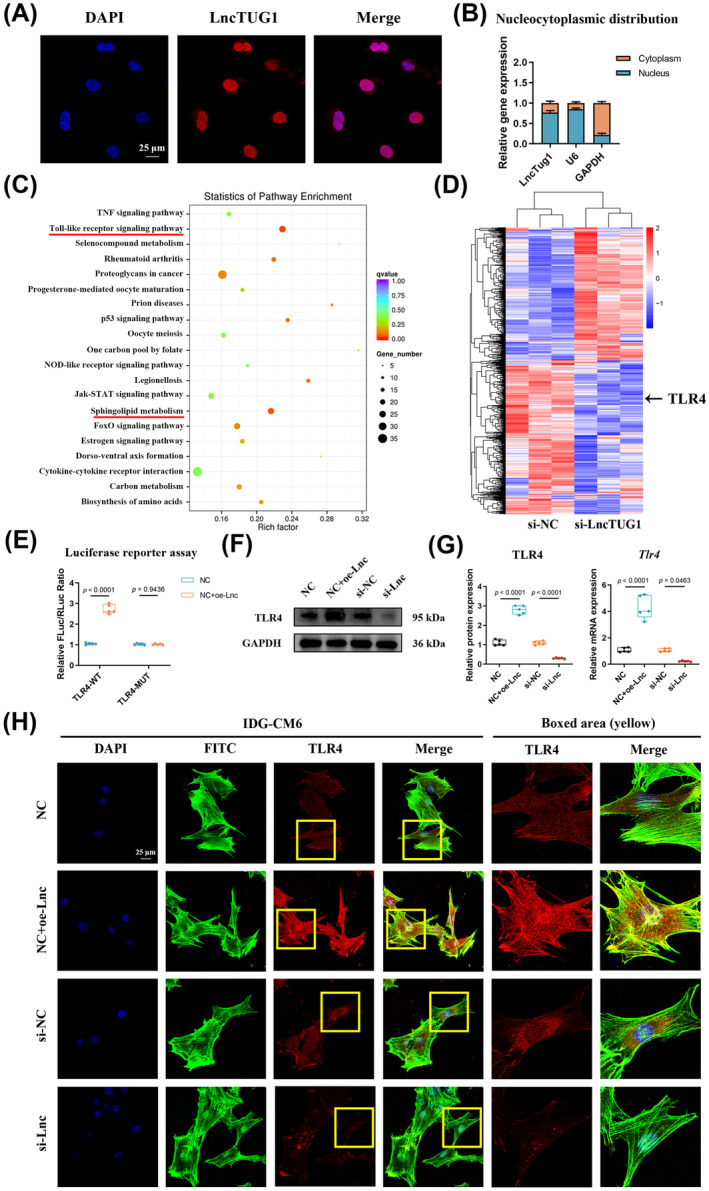
Long non‐coding ribonucleic acid (lncRNA) taurine‐upregulated gene 1 (LncTUG1) positively correlates with the expression of Toll‐like receptor 4 (TLR4). (A,B) The subcellular location of LncTUG1 in IDG‐CM6 cells was examined by the RNA FISH and subcellular fractionation assays (U6 and GAPDH were served as the nuclear and cytoplasmic controls, respectively). Scale bar = 25 μm. (C) The Kyoto Encyclopedia of Genes and Genomes (KEGG) analysis showed significantly enriched signalling pathways in IDG‐CM6 after knockdown of LncTUG1. (D) Heatmap analysis of differentially expressed genes (DEGs) in IDG‐CM6 after knockdown of LncTUG1. (E) Luciferase reporter assay confirmed the facilitating effect of LncTUG1 on TLR4 promotor activity. (F,G) Gain‐ and loss‐of‐function experiments were performed to examine the protein and mRNA levels of TLR4 in IDG‐CM6 cells using Western blot and qRT‐PCR analyses. GAPDH was used as a loading control. (H) Representative immunofluorescence images of TLR4 level in IDG‐CM6 cells. Cytoskeleton, green; TLR4, red; nuclei, blue. Scale bar = 25 μm. Statistical comparison was performed using one‐way analysis of variance with Tukey's post hoc test. *p* < 0.05 was considered statistically significant. At least five independent experiments were conducted.

Since the regulatory roles of lncRNAs are associated with their subcellular localization,^30^ we performed the FISH and subcellular fractionation assays illustrating that LncTUG1 was mainly distributed in the nuclear fraction (Figure 2A,B), which suggested that LncTUG1 might regulate the transcription of targeted genes.^33^ Based on this finding, we further investigated the downstream gene targeted by LncTUG1 during stress‐induced cementocyte mineralization.

We first performed RNA transcriptome sequencing following LncTUG1 knockdown. The KEGG analysis showed the enriched top 20 signalling pathways (Figure 2C). Among them, the TLR signalling pathway and the sphingolipid metabolism were most significantly enriched, with both being previously documented as active signalling cascades in osteogenesis and mineralization.^23^, ^34^ As the upstream regulator of sphingolipid metabolism,^35^ the TLR signalling pathway was selected as a candidate axis downstream of LncTUG1 during stress‐induced cementocyte mineralization. Further heatmap analysis revealed that TLR4, a critical component of TLR signalling and actively involved in regulating periodontium osteogenic mineralization,^23^, ^24^ was significantly downregulated by LncTUG1 knocked‐down (Figure 2D). Thus, we focused on this gene and conducted the luciferase reporter assay to examine the association between LncTUG1 and TLR4. The results suggested that LncTUG1 overexpression promoted the reporter luciferase activity of TLR4; however, this increase was cancelled when the promotor region of TLR4 was mutated (Figure 2E). Moreover, qRT‐PCR, Western blot, and immunofluorescence showed that overexpression of LncTUG1 could increase, while LncTUG1 knockdown could suppress the expression of TLR4 (Figure 2F–H). Meanwhile, upregulation or downregulation of TLR4 failed to influence the mRNA level of LncTUG1 (Figure S2).

### 3.4 TLR4 also played a negative role in stress‐induced mineralization of IDG‐CM6 cells

**FIGURE 3:**
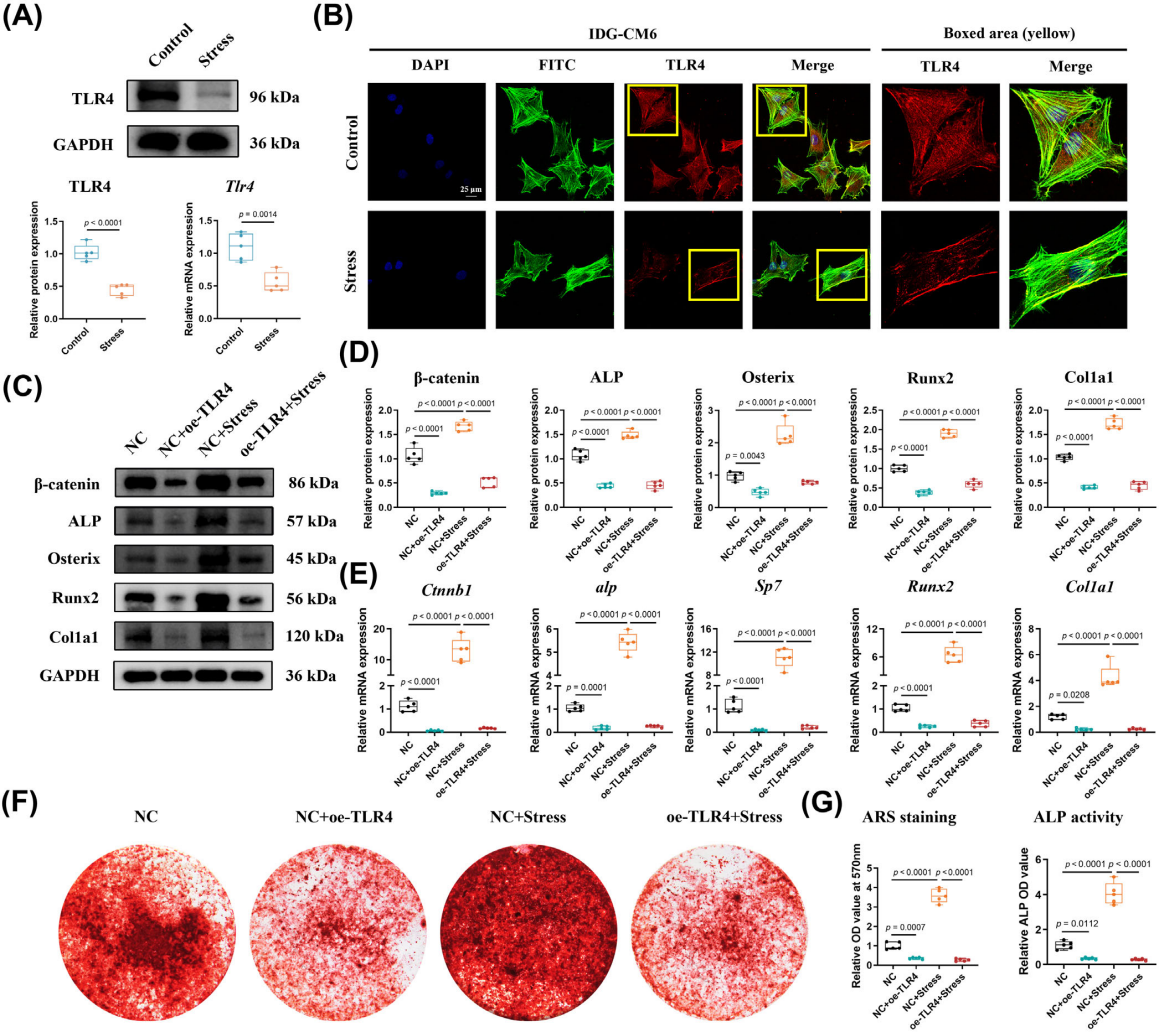
Toll‐like receptor 4 (TLR4) plays a negative role in stress‐induced mineralization of IDG‐CM6 cells. (A) The expression of TLR4 in IDG‐CM6 cells was assessed by western blot and qRT‐PCR analyses using GAPDH as a loading control and the corresponding quantitative analysis was performed. (B) Representative immunofluorescence images of TLR4 level in IDG‐CM6 cells under desirable compression force. Cytoskeleton, green; TLR4, red; nuclei, blue. Scale bar = 25 μm. (C,D) The expressions of β‐catenin, ALP, Osterix, Runx2, and Col1a1 in IDG‐CM6 cells, after overexpression of TLR4 and/or application of desirable compression force, were assessed by western blot analysis using GAPDH as a loading control and the corresponding quantitative analysis was performed. (E) The qRT‐PCR analysis was performed to examine the mRNA levels of *Ctnnb1*, *alp*, *Sp7*, *Runx2*, and *Col1a1* in IDG‐CM6 cells. (F,G) Representative images and quantitative analysis of Alizarin Red S (ARS) staining as well as the activity of ALP were conducted in IDG‐CM6 cells. Statistical comparison was performed using one‐way analysis of variance with Tukey's post hoc test. *p* < 0.05 was considered statistically significant. At least five independent experiments were conducted.

TLR4, predicted and testified as the critical gene associated with LncTUG1, was recently considered a novel regulator of osteogenic mineralization of the periodontium.^23^, ^24^ Thus, we hypothesized that TLR4 might actively participate in stress‐induced cementocyte mineralization. The results of qRT‐PCR, Western blot, and immunofluorescence indicated that the level of TLR4 in IDG‐CM6 cells under favourable compressive force was significantly declined compared with the control group (Figure 3A,B). To further identify the regulatory role of TLR4 in mechanical force‐induced mineralization of IDG‐CM6 cells, plasmids were used to overexpress TLR4, and the efficiency was verified by qRT‐PCR, Western blot, and immunofluorescence analyses (Figure S3). The results of qRT‐PCR, Western blot, and ARS staining indicated that the expressions of β‐catenin, ALP, Osterix, Runx2, and Col1a1 decreased significantly after the overexpression of TLR4 (Figure 3C–E). Moreover, the overexpression of TLR4 partially reversed the promoting effect of mechanical forces on those mineralization‐associated genes (Figure 3C–E). Figure 3F,G consistently showed similar trends of mineralized nodule formation and the activity of ALP.

### 3.5 LncTUG1‐regulated stress‐induced mineralization of IDG‐CM6 cells via targeting TLR4

**FIGURE 4:**
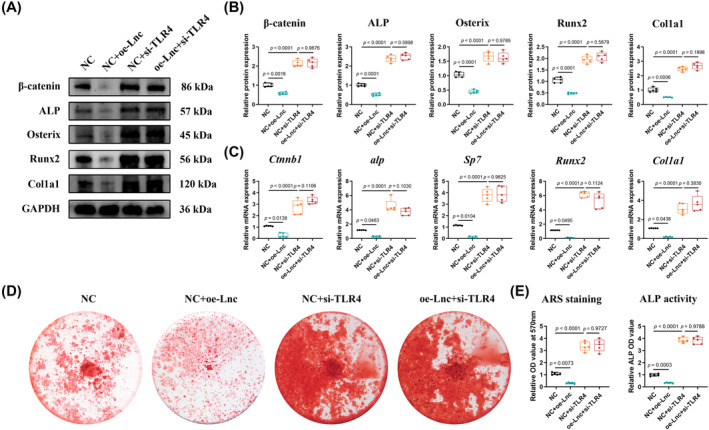
Long non‐coding ribonucleic acid (lncRNA) taurine‐upregulated gene 1 (LncTUG1) regulates stress‐induced mineralization of IDG‐CM6 cells via targeting Toll‐like receptor 4 (TLR4). (A,B) The expressions of β‐catenin, ALP, Osterix, Runx2, and Col1a1 in IDG‐CM6 cells, after overexpression of LncTUG1 and/or knockdown of TLR4, were assessed by western blot analysis using GAPDH as a loading control and the corresponding quantitative analysis was performed. (C) The qRT‐PCR analysis was performed to examine the mRNA levels of *Ctnnb1*, *alp*, *Sp7*, *Runx2* and *Col1a1* in IDG‐CM6 cells. (D,E) Representative images and quantitative analysis of Alizarin Red S (ARS) staining as well as the activity of ALP were conducted in IDG‐CM6 cells. Statistical comparison was performed using one‐way analysis of variance with Tukey's post hoc test. *p* < 0.05 was considered statistically significant. At least five independent experiments were conducted.

Since the overexpression of either LncTUG1 or TLR4 could interfere with the mineralization of IDG‐CM6 cells (Figures 1 and 3), and the regulatory effect of LncTUG1 on TLR4 has been verified (Figures 2 and S2), we further determined whether LncTUG1 regulates stress‐induced mineralization of IDG‐CM6 cells by targeting TLR4. The siRNA was used to knock down TLR4 expression, and the efficiency was verified by qRT‐PCR, Western blot, and immunofluorescence analyses (Figure S4). The results showed that under favourable compression force, the mRNA and protein levels of β‐catenin, ALP, Osterix, Runx2, and Col1a1 increased significantly when TLR4 was knocked down (Figure 4A–C). Furthermore, the rescue experiments showed that the knockdown of TLR4 could offset the inhibitory effect of LncTUG1 on those mineralization‐associated genes (Figure 4A–C). Similar trends were also observed in ARS staining and ALP activity examination assays (Figure 4D,E). These findings suggested that LncTUG1 could regulate stress‐induced mineralization of IDG‐CM6 cells by targeting TLR4.

### 3.6 SphK1 served as a real target for TLR4 in stress‐induced mineralization of IDG‐CM6 cells

**FIGURE 5:**
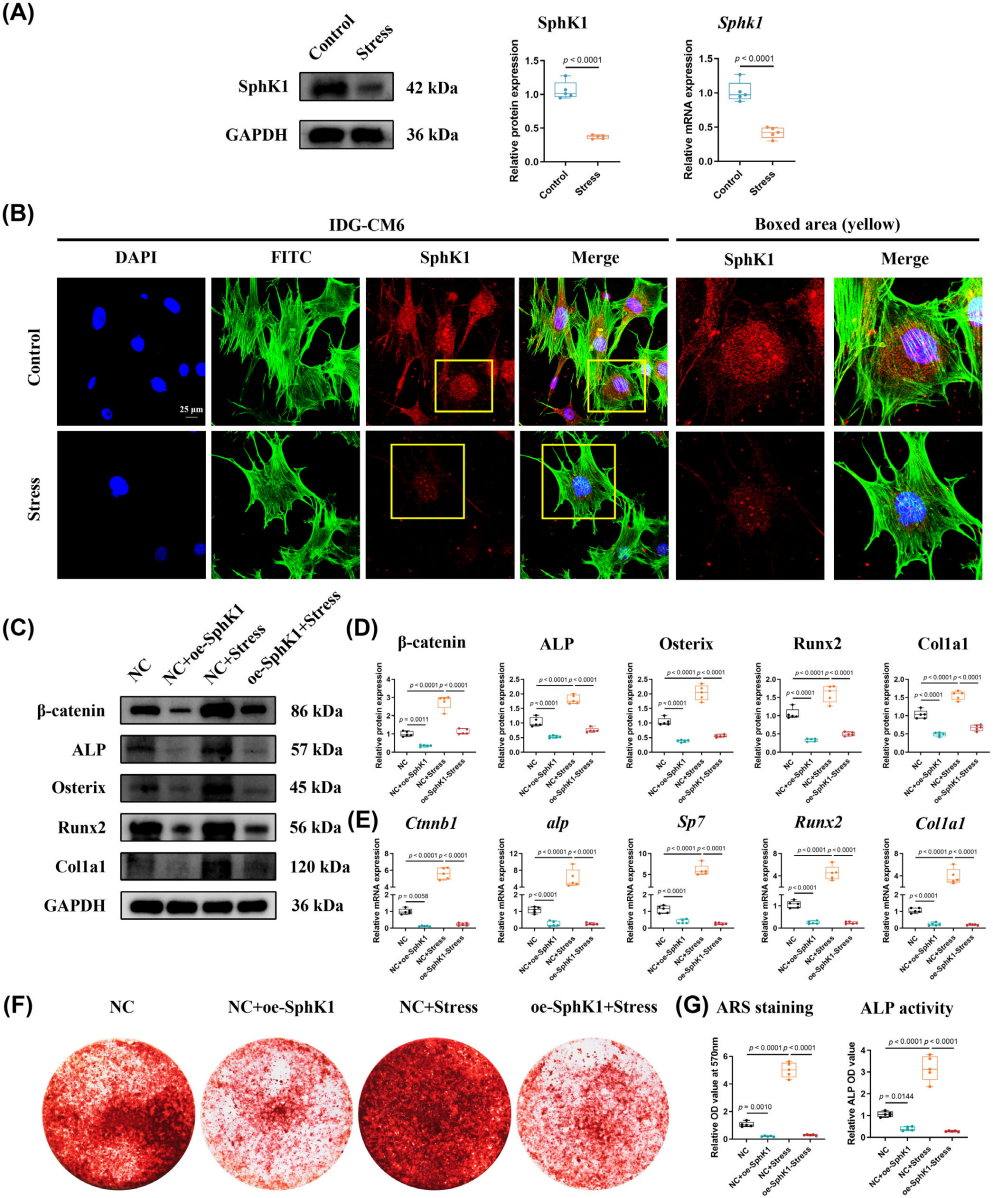
SphK1 plays a negative role in stress‐induced mineralization of IDG‐CM6 cells. (A) The expression of SphK1 in IDG‐CM6 cells was assessed by western blot and qRT‐PCR analyses using GAPDH as a loading control and the corresponding quantitative analysis was performed. (B) Representative immunofluorescence images of SphK1 level in IDG‐CM6 cells under desirable compression force. Cytoskeleton, green; SphK1, red; nuclei, blue. Scale bar = 25 μm. (C,D) The expressions of β‐catenin, ALP, Osterix, Runx2, and Col1a1 in IDG‐CM6 cells, after overexpression of SphK1 and/or application of desirable compression force, were assessed by western blot analysis using GAPDH as a loading control and the corresponding quantitative analysis was performed. (E) The qRT‐PCR analysis was performed to examine the mRNA levels of *Ctnnb1*, *alp*, *Sp7*, *Runx2*, and *Col1a1* in IDG‐CM6 cells. (F,G) Representative images and quantitative analysis of Alizarin Red S (ARS) staining as well as the activity of ALP were conducted in IDG‐CM6 cells. Statistical comparison was performed using one‐way analysis of variance with Tukey's post hoc test. *p* < 0.05 was considered statistically significant. At least five independent experiments were conducted.

Our preliminary in vivo study demonstrated that S1P signalling could influence the levels of mineralization‐associated factors (Runx2 and OPN) in force‐loaded cementocytes.^17^ Based on this preliminary finding, we performed an in vitro study, and the results of qRT‐PCR, Western blot, and immunofluorescence revealed that the levels of SphK1, the predominant isoform that catalyses S1P,^36^, ^37^ in IDG‐CM6 cells under favourable compressive force decreased significantly compared with the control group (Figure 5A,B). We conducted further intervention experiments. First, the overexpression of SphK1 was confirmed by qRT‐PCR, Western blot, and immunofluorescence analyses (Figure S5). Based on this, our findings demonstrated that the expressions of β‐catenin, ALP, Osterix, Runx2, and Col1a1 decreased significantly after the overexpression of SphK1 (Figure 5C–E). Moreover, the overexpression of SphK1 partially reversed the promoting effects of mechanical forces on those mineralization‐associated genes (Figure 5C–E). Figure 5F,G consistently show similar trends of mineralized nodule formation and the activity of ALP.

**FIGURE 6:**
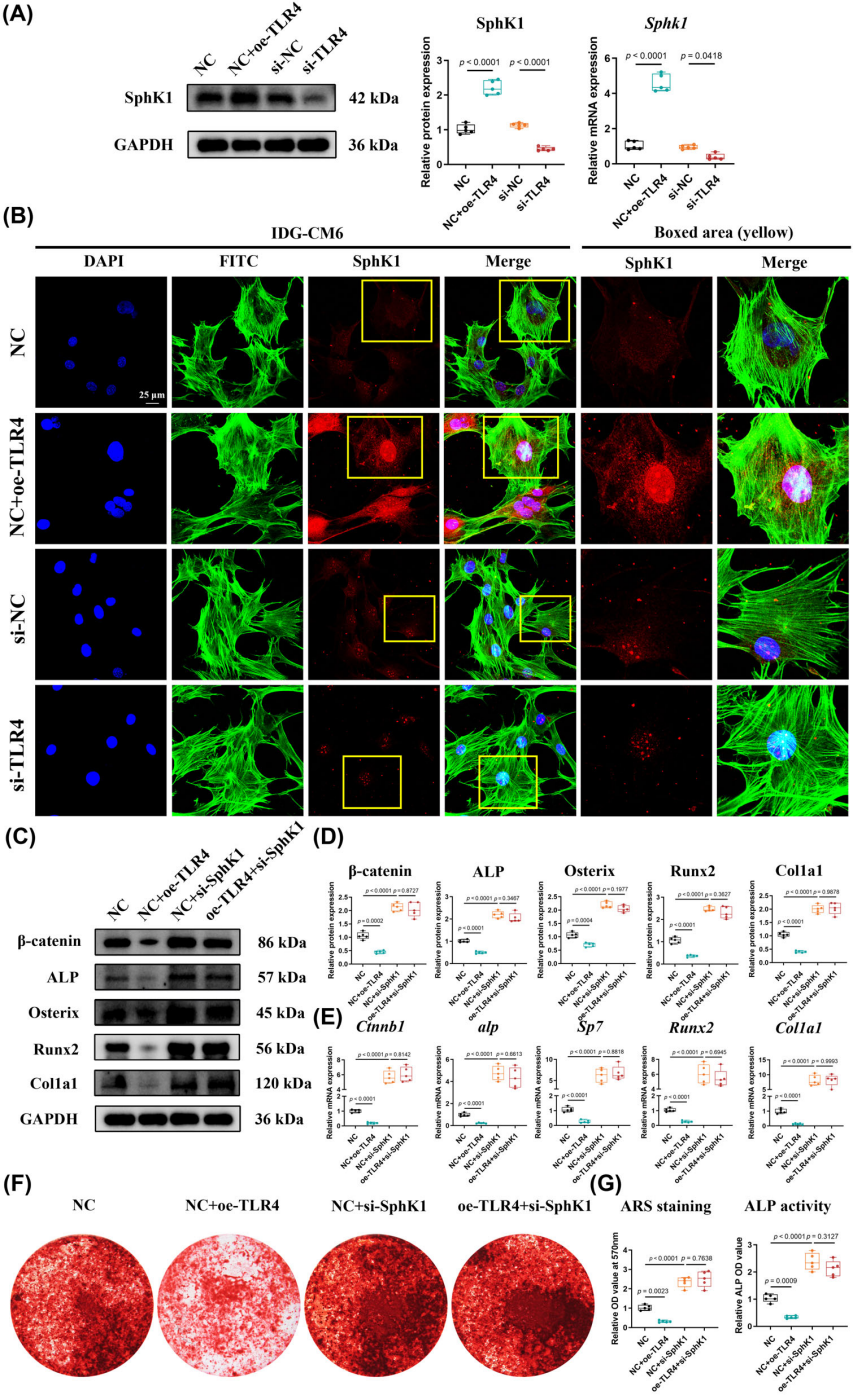
SphK1 is a bona fide target for Toll‐like receptor 4 (TLR4) in stress‐induced mineralization of IDG‐CM6 cells. (A) Gain‐ and loss‐of function experiments were performed to examine the protein and mRNA levels of SphK1 in IDG‐CM6 cells using Western blot and qRT‐PCR analyses. GAPDH was used as a loading control. (B) Representative immunofluorescence images of SphK1 level in IDG‐CM6 cells. Cytoskeleton, green; SphK1, red; nuclei, blue. Scale bar = 25 μm. (C,D) The expressions of β‐catenin, ALP, Osterix, Runx2, and Col1a1 in IDG‐CM6 cells, after overexpression of LncTUG1 and/or knockdown of TLR4, were assessed by western blot analysis using GAPDH as a loading control and the corresponding quantitative analysis was performed. (E) The qRT‐PCR analysis was performed to examine the mRNA levels of *Ctnnb1*, *alp*, *Sp7*, *Runx2*, and *Col1a1* in IDG‐CM6 cells. (F,G) Representative images and quantitative analysis of Alizarin Red S (ARS) staining as well as the activity of ALP were conducted in IDG‐CM6 cells. Statistical comparison was performed using one‐way analysis of variance with Tukey's post hoc test. *p* < 0.05 was considered statistically significant. At least five independent experiments were conducted.

Previous studies have reported that SphK1 could be regulated by TLR4.^35^ Our findings showed that both TLR4 and SphK1 played critical roles in stress‐induced mineralization of IDG‐CM6 cells (Figures 3 and 5). Thus, we first performed qRT‐PCR, Western blot, and immunofluorescence assays to identify the regulatory relationship between TLR4 and SphK1. The efficiency of the siRNA we used to knock down SphK1 expression was confirmed by qRT‐PCR, Western blot, and immunofluorescence analyses (Figure S6). The results of qRT‐PCR, Western blot, and immunofluorescence assays indicated that the overexpression of TLR4 could enhance while the knockdown of TLR4 could suppress SphK1 levels (Figure 6A,B). Furthermore, the rescue experiments showed that the knockdown of SphK1 could offset the inhibitory effect of TLR4 on those mineralization‐associated genes (Figure 6C–E). Similar trends were also observed in ARS staining and ALP activity examination assays (Figure 6F,G). These findings suggested that SphK1 is a real target for TLR4 in stress‐induced mineralization of IDG‐CM6 cells.

### 3.7 LncTUG1‐regulated TLR4 expression at the transcriptional level through direct binding with PU.1

**FIGURE 7:**
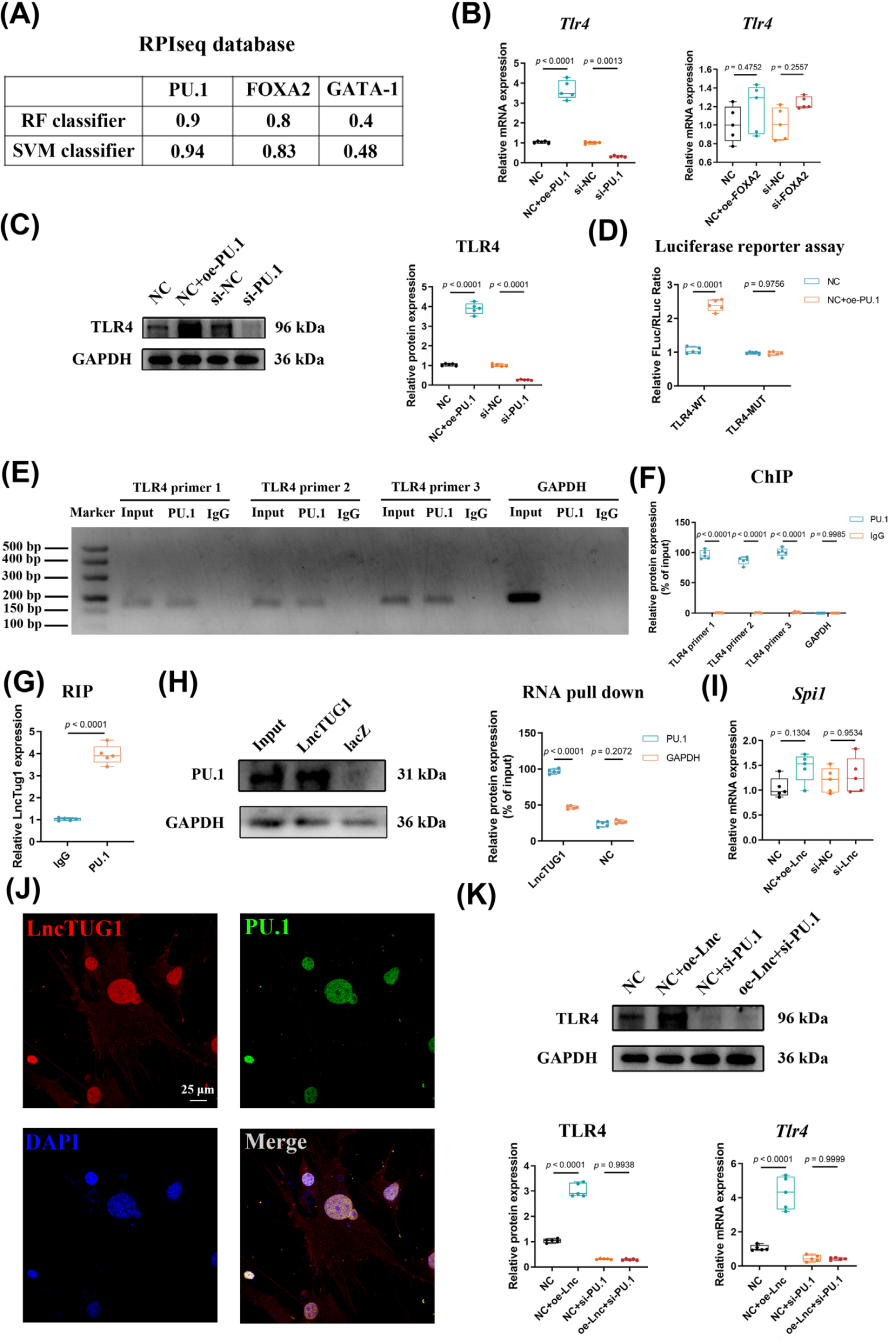
Long non‐coding ribonucleic acid (lncRNA) taurine‐upregulated gene 1 (LncTUG1) regulates Toll‐like receptor 4 (TLR4) expression at the transcriptional level through directly binding with PU.1. (A) The possibility of LncTUG1 interacting with PU.1, FOXA2, and GATA‐1 was identified using the RPIseq database. Random forest (RF) and support vector machine (SVM) models with probabilities higher than 0.5 were considered positive. (B) The Effect of PU.1 and FOXA2 knockdown on the mRNA level of *Tlr4* by qRT‐PCR. (C) Gain‐ and loss‐of‐function experiments were performed to examine the protein level of TLR4 in IDG‐CM6 cells using Western blot analysis. The intervention of PU.1 was also verified and quantified by Western blot analysis. (D) Luciferase reporter assay confirmed the interaction between PU.1 and the promotor region of TLR4. (E,F) PU.1 binds to the TLR4 promoter indicated by ChIP analysis as well as the corresponding quantitative analysis in IDG‐CM6 cells. IgG was served as a negative control. (G,H) RIP and RNA pull‐down experiments confirmed the interaction between LncTUG1 and PU.1. The lacZ probe was used as control of the RNA pull‐down assay. (I) The mRNA level of *Spi1* (PU.1) following overexpression or knockdown of LncTUG1 assessed by qRT‐PCR analysis. (J) Co‐localization analysis by the RNA FISH assay of LncTUG1 (red) combined with the immunofluorescence detection of PU.1 (green) in IDG‐CM6 cells. Scale bar = 25 μm. (K) The expression of TLR4 in IDG‐CM6 cells, after overexpression of LncTUG1 and/or knockdown of PU.1, was assessed by western blot and qRT‐PCR analyses using GAPDH as a loading control and the corresponding quantitative analysis was performed.

Given the high affinity of LncTUG1 for the promotor region of TLR4 and since one of the ways in which lncRNAs function is by binding to TFs to regulate mRNA levels,^38^ we hypothesized that LncTUG1 might interact with specific TFs to regulate TLR4 expression. We performed a sequence‐based TFs prediction using the PROMO site (https://alggen.lsi.upc.es/cgi‐bin/promo_v3/promo/promoinit.cgi?dirDB=TF_8.3) to clarify the potential TFs bound to LncTUG1 and identified a cluster of candidate TFs that interact with LncTUG1. Among the candidates, spleen focus‐forming virus proviral integration oncogene (PU.1/SPI1), Forkhead box protein A2 (FOXA2), and GATA binding protein 1 (GATA‐1) have been reported to possibly regulate the expression of TLR4.^39^, ^40^, ^41^, ^42^ Then, we used another website (http://pridb.gdcb.iastate.edu/RPISeq/index.html) to predict the probability of LncTUG1 binding with the three TFs. The results revealed that as the random forest and support vector machine values suggested (>0.5), two of those three TFs (PU.1 and FOXA2) had a strong likelihood of binding to LncTUG1 (Figure 7A). Thus, we selected PU.1 and FOXA2 for further analyses.

The qRT‐PCR analysis indicated that overexpression and knockdown of PU.1 could, respectively, upregulate and downregulate the expression of TLR4, whereas overexpression and knockdown of FOXA2 did not affect TLR4 (Figure 7B). Accordingly, we focused on PU.1 and conducted the Western blot and luciferase reporter assays to verify whether PU.1 could enhance the promotor activity and protein level of TLR4 (Figure 7C,D). Moreover, the ChIP assay was performed to explore the interaction between PU.1 and TLR4. Three randomly designed primers of promotor regions of TLR4 were examined, and the results demonstrated that PU.1 could bind to the TLR4 promoter region (Figure 7E,F). Moreover, the RIP assay confirmed that PU.1 could directly bind to LncTUG1 (Figure 7G). The RNA pull‐down assay consistently verified the interaction between PU.1 and LncTUG1 (Figure 7H). Meanwhile, the co‐localization of PU.1 with LncTUG1 was observed by confocal microscopy. The mRNA level of *Spi1* (PU.1) was not affected by overexpression or knockdown of LncTUG1 (Figure 7I,J), which, combined with the findings above, indicated that LncTUG1 could specifically bind to PU.1. Furthermore, we investigated the regulatory effects of LncTUG1 on TLR4 by binding to PU.1 and the results of gain‐ and loss‐of‐function experiments showed that the overexpression of LncTUG1 failed to promote the mRNA and protein levels of TLR4 when PU.1 was knocked down (Figure 7K). To further confirm the above findings, we used the catRAPID website (http://service.tartaglialab.com/page/catrapid_group) to predict potential binding regions between LncTUG1 and PU.1. The candidate sequence of RNA‐binding motifs of PU.1 was GGCTTTCCTCTGGAG. The results of luciferase reporter assays showed no changes in the TLR4 promotor activity after the mutation of the selected motif (Figure S7).

### 3.8 The TLR4/SphK1 axis was involved in favourable orthodontic force‐induced cementocyte mineralization during OTM of rats

**FIGURE 8:**
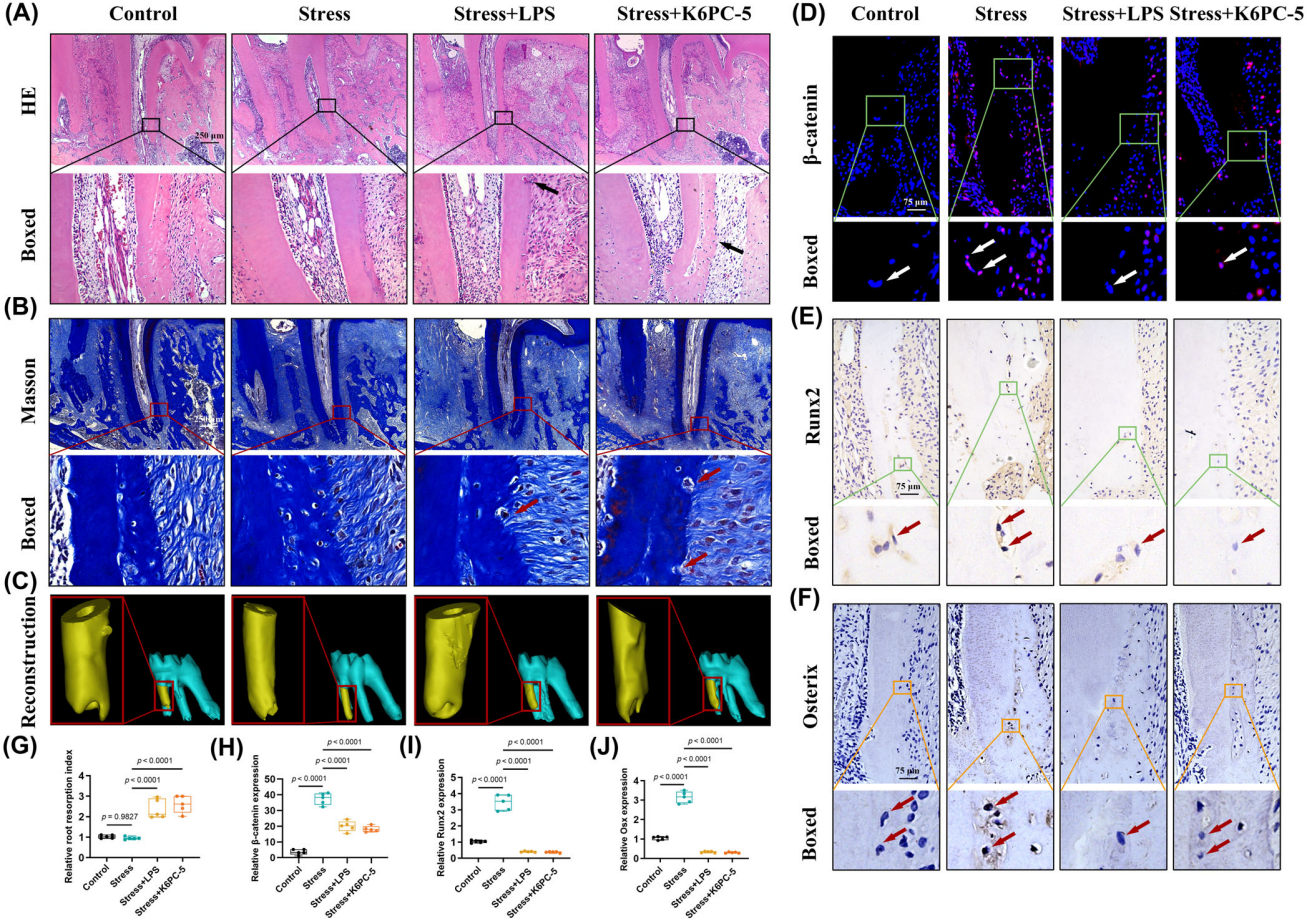
The Toll‐like receptor 4 (TLR4)/SphK1 axis is involved in orthodontic force‐induced cementocytes mineralization during orthodontic tooth movement (OTM). (A) Representative haematoxylin and eosin (HE) staining images of the distobuccal roots of M1. The black arrow indicates the resorption pits. Scale bar = 250 μm. (B) Representative Masson's trichrome staining images of the distobuccal roots of M1. The red arrow indicates the resorption pits. Scale bar = 250 μm. (C) The three‐dimensional reconstruction of M1 using Mimics 21.0. The red box indicated the distobuccal root of M1. (D) Representative immunofluorescence images of β‐catenin (red) at the apical third region of distobuccal roots. The white arrow indicates the cementocytes at the interested area. Scale bar = 75 μm. (E) Representative immunohistochemical images of Runx2 at the apical third region of distobuccal roots. The red arrow indicates the cementocytes at the interested area. Scale bar = 75 μm. (F) Representative immunohistochemical images of Osterix at the apical third region of distobuccal roots. The red arrow indicates the cementocytes at the interested area. Scale bar = 75 μm. (G) The distobuccal roots of M1 were scanned by microcomputed tomography and reconstructed to calculate the root resorption volumes. The resorption index was normalized to the control group. (H) Semi‐quantitative analysis of β‐catenin expression. (I) Semi‐quantitative analysis of Runx2 positive staining. (J) Semi‐quantitative analysis of Osterix positive staining. Statistical comparison was performed using one‐way analysis of variance with Tukey's post hoc test. *p* < 0.05 was considered statistically significant. At least five independent experiments were conducted.

Consistent with the morphology of the root surface shown by HE and Masson's trichrome staining (Figure 8A,B), the volume of the resorption lacunae, analysed by Mimics, increased significantly in both the stress + LPS and stress + K6PC‐5 groups compared with the stress group (Figure 8C,G). Immunofluorescence staining and the corresponding analysis showed a weaker positive intensity of β‐catenin in both the stress + LPS and stress + K6PC‐5 groups compared with the stress group (Figure 8D,H). Consistently, immunohistochemical staining and semi‐quantitative analysis showed weaker positive staining of Runx2 and Osterix in both the stress+ LPS and stress + K6PC‐5 groups compared with the stress group (Figure 8E,F,I,J). Overall, these findings suggested the involvement of TLR4/SphK1 signalling in favourable orthodontic force‐induced cementocyte mineralization during OTM in rats.

## 4 DISCUSSION

This study showed that favourable force could induce the mineralization of IDG‐CM6 cells (cementocytes). Moreover, the RNA‐seq following the mechanical loading of IDG‐CM6 cells helped us focus on LncTUG1 and its regulatory role in cementocyte mineralization. After verifying the nuclear localization of LncTUG1 and performing RNA‐seq after LncTUG1 knockdown, we targeted TLR4/SphK1 as the key downstream signalling of LncTUG1 and confirmed that LncTUG1 modulated stress‐induced cementocyte mineralization via the TLR4/SphK1 axis. The in vivo experiment also confirmed the crucial role of TLR4/SphK1 signalling in favourable orthodontic force‐induced cementocyte mineralization during OTM. Mechanistically, our findings demonstrated that LncTUG1 could upregulate the transcription levels of TLR4 through direct binding to PU.1, the specific TF that recognizes the promotor region of TLR4.

OTM is a highly coordinated biomechanical response to favourable orthodontic force with active remodelling of alveolar bone but little root resorption.^43^, ^44^ Future studies should elucidate whether the antiresorptive properties of root derive from cellular antiresorptive signalling associated with resident cells in periodontal tissues.^45^ Recently, many studies have focused on the regulatory role of periodontal cellular mechanotransduction in root resistance to resorption during OTM.^44^, ^46^ For instance, a previous study demonstrated that orthodontic force‐loaded periodontal ligament cells (PDLCs) could alleviate root resorption by inhibiting CXCL12 during OTM.^44^ In addition, compressive stress could regulate the mineralization capability of cementoblasts by interfering with the autophagic process, influencing root resistance to resorption.^46^ However, a limitation common to the above studies might be that the cells studied were all components of the periodontal ligament instead of the root cementum, the barrier, and the most important part of the root to resist resorption.^3^, ^4^, ^17^ Additionally, since cementocytes and osteocytes have recently been shown to share similar morphological and functional characteristics,^3^, ^4^, ^47^ logically, we proposed that cementocytes are dynamic components in cementum comparable to osteocytes in the skeleton. Thus, our study focused on the mechanotransduction of cementocytes, the primary cellular components of root cementum, and demonstrated that a favourable compressive force could promote cementocyte mineralization, consistent with the phenomenon observed in clinical orthodontics.^48^ These findings laid the foundation for further exploration of regulatory mechanisms of cemenctocytes' mechanotransduction effect on root resistance to resorption.

The investigation into lncRNA‐regulated mineralization and osteogenesis of cells in periodontal tissues during OTM is still in its infancy, providing less extensive information due to the complicated mechanisms of the functions of lncRNAs and their multiple targets.^48^ Based on the first part of the findings above, we further explored whether and how lncRNAs regulate stress‐induced cementocyte mineralization. In particular, differentially expressed lncRNAs were first screened out as the potential targets to modulate cementocyte mineralization. The GO analysis indicated that genes co‐expressed with those lncRNAs were highly associated with ossification, cell differentiation, and responses to mechanical stimuli, suggesting that lncRNAs might actively regulate stress‐induced cementocyte mineralization via certain targeted genes.^49^ To identify the most likely lncRNA regulating this mineralization process, we selected the top significantly altered lncRNAs co‐expressed with genes, and eight lncRNAs met the conditions. Furthermore, qRT‐PCR results verified that only six of eight lncRNAs changed significantly after applying the compressive force, of which LncTUG1 was the most significantly altered one. Accordingly, we focused on LncTUG1 and performed gain‐of‐function studies to evaluate its role in regulating stress‐induced cementocyte mineralization. A previous study revealed the role of LncTUG1 in osteoarthritis by modulating apoptosis and inflammatory response of chondrocytes.^50^ Meanwhile, the regulatory effects of LncTUG1 on bone fracture recovery have also been demonstrated recently.^51^ Both these studies have effectively validated LncTUG1's active involvement in maintaining the homeostasis of bone and cartilage tissues. Additionally, as our investigation focused on mineralized tissues, the results indicated that the promotion of cementum mineralization by compressive forces was significantly counteracted by the overexpression of LncTUG1. At the same time, it was notably enhanced by the LncTUG1 knockdown. Altogether, it is reasonable to introduce LncTUG1 as a critical regulator in stress‐induced cementocyte mineralization. However, the specific targeted genes of LncTUG1 during its regulation of stress‐induced cementocyte mineralization remain to be identified.

To investigate the targeted genes of LncTUG1 during stress‐induced cementocyte mineralization after verifying its nuclear localization, we further conducted RNA sequencing to determine the possible genes targeted by LncTUG1 in regulating stress‐induced cementocyte mineralization. Through the KEGG analysis following the LncTUG1 knockdown, we focused on the TLR signalling. The heatmap analysis of DEGs led us to introduce TLR4 as the key factor downstream of LncTUG1. Furthermore, luciferase reporter assays and gain‐/loss‐of‐function experiments confirmed that LncTUG1 could enhance the promotor activity as well as the mRNA and protein levels of TLR4. Several lines of evidence have recently documented that TLR4 could inhibit the osteogenic differentiation of PDLCs and PDLSCs, which might further aggravate Pg‐LPS‐induced periodontitis and alveolar bone loss.^23^, ^24^ As one of the essential components of periodontal tissues and the ‘cousin’ of bone, cementum mineralization was also found to be highly related to TLR4 expression. Our findings suggested that TLR4 reversed favourable compressive force‐induced cementocyte mineralization. Since TLR4 was the key targeted gene of LncTUG1 and both of them were actively involved in stress‐induced cementocyte mineralization, we speculated that LncTUG1 might regulate stress‐induced cementocyte mineralization via targeting TLR4, and our findings confirmed this speculation.

Another most significantly enriched signalling in the KEGG analysis of LncTUG1 knockdown, sphingolipid metabolism, was also investigated in this study. Previous research has demonstrated the critical role of the SphK1/S1P axis in regulating bone metabolism.^52^, ^53^, ^54^, ^55^ Specifically, SphK1/S1P could respond to mechanical stimuli and subsequently modulate the mineralization‐associated genes of osteocytes, such as RANKL and OPG.^55^ As the ‘cousin’ of osteocytes,^3^ cementocytes could also sense and transduce the mechanical signals and regulate the mineralization‐associated genes through S1P signalling, as reported in our preliminary in vivo research.^17^ Our in vitro study consistently confirmed the regulatory role of SphK1, the main kinase catalysing S1P,^17^ in stress‐induced cementocyte mineralization. Our preliminary study verified that SphK1 was a key regulator of the mineralization‐related factor, β‐catenin, consistent with previous studies.^52^, ^54^ Also, actively involved in regulation of β‐catenin,^20^, ^21^, ^55^ TLR4 might be the potential upstream regulator of SphK1/S1P, documented in previous studies,^35^, ^56^, ^57^ in stress‐induced cementocyte mineralization. Our gain‐/loss‐of‐function assays revealed that TLR4 could positively regulate the expression of SphK1, and the TLR4/SphK1 axis was a key signalling cascade in regulating stress‐induced cementocyte mineralization. Consistent with this finding, our in vivo data also confirmed the critical role of the TLR4/SphK1 axis in orthodontic force‐induced cementocyte mineralization during OTM.

As mainly distributed in the nuclei of the IDG‐CM6 cells, LncTUG1 was speculated to be a transcriptional regulator that exerted its main biological effects by binding to targeted TFs.^58^, ^59^ To further investigate the specific mechanism by which LncTUG1 modulates the expression of TLR4, we used bioinformatics analysis to predict potential TFs that might interact with LncTUG1. The results screened out PU.1 and FOXA2 as candidates. Further gain‐/loss‐of‐function experiments suggested that only PU.1 had significant regulatory effects on TLR4 expression, and the subsequent luciferase reporter assay confirmed the interaction between PU.1 and TLR4. Based on the above, we focused on PU.1 for further analyses. PU.1 has been extensively documented as an essential regulator of bone formation and mineralization.^60^, ^61^, ^62^ According to previous studies, PU.1 is located on the promotor region of TLR4^61^, ^62^ and further triggers the release of inflammatory cytokines that contribute to bone loss.^44^, ^63^ Our ChIP assay consistently confirmed the interaction between PU.1 and TLR4 promotor region in the IDG‐CM6 cells. Meanwhile, RIP, RNA pull‐down, and luciferase reporter assays showed that PU.1 could directly bind to LncTUG1, which was also supported by the co‐localization experiment using immunofluorescence. Consistent with our results, a previous study also indicated a direct role for PU.1 in regulating TLR4 transcription through rapid alteration of transcription start sites (TSSs) and enhanced the recruitment of polymerase II to the proximal TLR4 promoter.^39^ Since TLR4 transcription initiates from the same conserved promoter region as PU.1 occupies, based on the above, we propose that LncTUG1 could interact with PU.1 to form a complex that recognizes the sites of TLR4 promotor region and is further recruited on it with subsequent accumulation of polymerase II to activate TLR4 transcription. Future studies could focus on the exact mechanisms of how the LncTUG1/PU.1 complex recognizes and activates TLR4 transcription.

**FIGURE 9:**
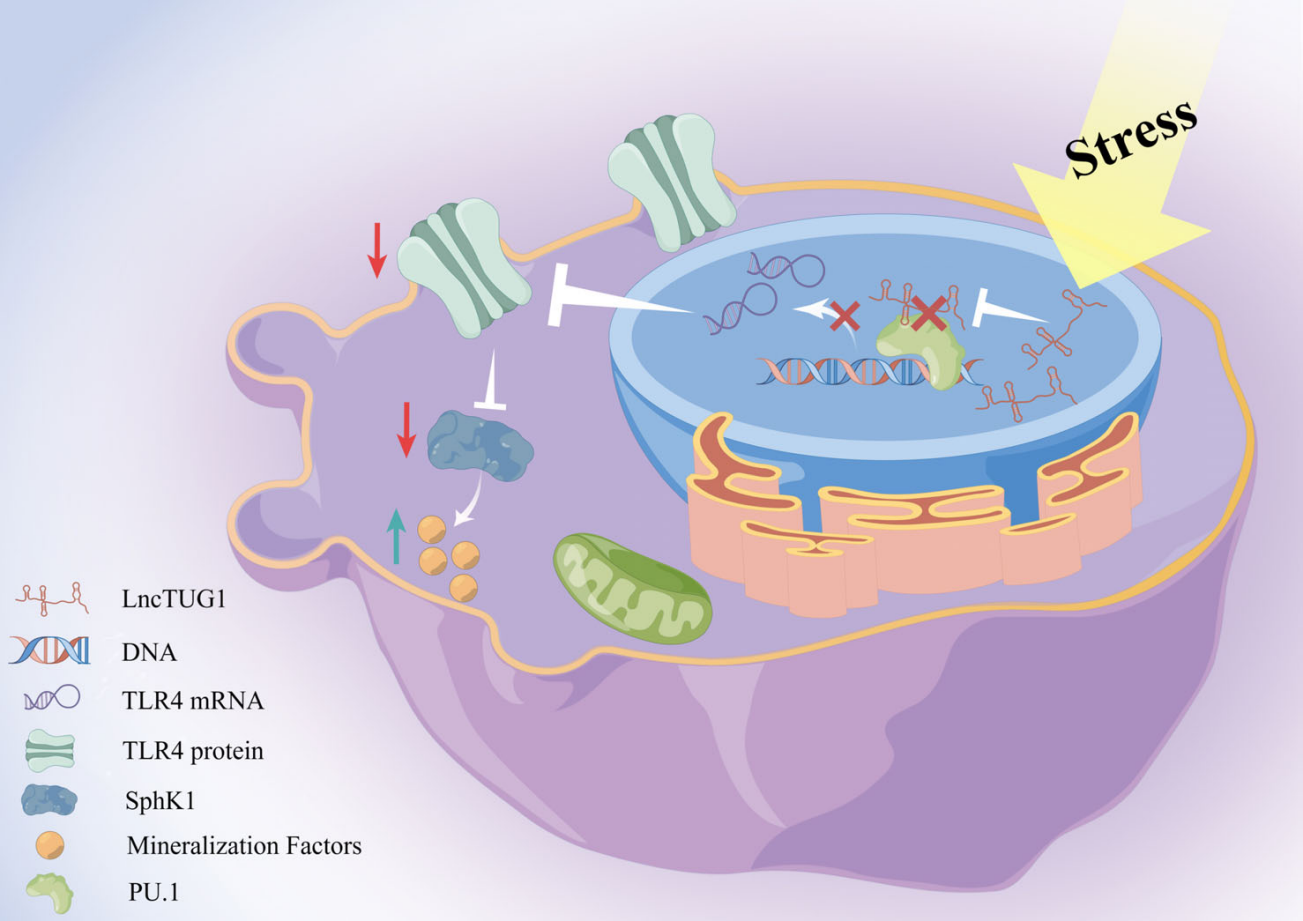
A schematic diagram illustrating the molecular mechanism of long non‐coding ribonucleic acid (lncRNA) taurine‐upregulated gene 1 (LncTUG1) in regulating stress‐induced cementocytes mineralization via PU.1/TLR4/SphK1 axis.

In summary, our study determined and validated the crucial role of LncTUG1 in stress‐induced cementocyte mineralization. LncTUG1‐modulated stress‐induced cementocyte mineralization via TLR4/SphK1 signalling. Mechanistically, LncTUG1 directly bound to PU.1, the specific TF of TLR4, and then the LncTUG1/PU.1 complex transcriptionally upregulated the expression of TLR4 in stress‐induced cementocyte mineralization (Figure 9). These findings might provide further insight into developing novel therapeutic strategies to protect the root from resorption during orthodontic treatment.

## AUTHOR CONTRIBUTIONS

**Han Wang:** Contributed to conception and design, data acquisition and interpretation, performed all statistical analyses, and drafted the article. **Tiancheng Li:** Contributed to design, data acquisition, and interpretation. **Yukun Jiang:** Contributed to data interpretation. **Shuo Chen:** Contributed to data acquisition. **Zuping Wu:** Contributed to data interpretation. **Xinyi Zeng:** Contributed to data interpretation. **Kuan Yang:** Contributed to data acquisition. **Peipei Duan:** Contributed to conception, design, and critically revised the article. **Shujuan Zou:** Contributed to conception, design and critically revised the article. All authors gave their final approval and agree to be accountable for all aspects of the work.

## FUNDING INFORMATION

The authors disclosed receipt of the following financial support for the research, authorship, and/or publication of this article. This work was supported by the National Natural Science Foundation of China (NSFC, Nos. 82271017 and 82071150), Sichuan Science and Technology Program, China (Nos. 21ZDYF1874 and 2022YFS0117), and Angelalign Scientific Research Fund (No. 21H0900‐5). Thanks to Figdraw (www.figdraw.com) for the assistance in creating Figure 9.

## CONFLICT OF INTEREST STATEMENT

The authors have declared that no conflict of interest exists.

## Supporting information


**Data S1:** Supporting information.

## Data Availability

No publicly available data or shared data are cited. All original data supporting the conclusion of this study are available from the corresponding author on request.
