# Supplementary material for: Long non‐coding RNA LncTUG1 regulates favourable compression force‐induced cementocytes mineralization via PU.1/TLR4/SphK1 signalling
Source: Cell Prolif. 2024 Feb 6;57(6):e13604. doi: 10.1111/cpr.13604 (PMC11150144; doi:10.1111/cpr.13604)
Supplement: Supplementary file 1 — Data S1: Supporting information. [file CPR-57-e13604-s001.docx]

**Original Article:**

**Long non-coding RNA LncTUG1 regulates favorable compression force-induced cementocytes mineralization via PU.1/TLR4/SphK1 signaling**

**Running title:** **LncTUG1 regulates cementocytes mineralization**

**Han Wang^1^, Tiancheng Li^1,2^, Yukun Jiang^1^, Shuo Chen^1^,** **Zuping Wu^1,3^, Xinyi Zeng^1^, Kuan Yang^1^, Peipei Duan^1^, Shujuan Zou^1^**

**Appendix Table S1. Sequences of primers used in qRT-PCR**

| Gene | Forward primer sequence (5′–3′) | Reverse primer sequence (5′–3′) |
| --- | --- | --- |
| *Ctnnb1* | GCTGCTGTCCTATTCCGAATGTCTG | GGCACCAATGTCCAGTCCAAGATC |
| *Alpl* | TCATTCCCACGTTTTCACATTC | GTTGTTGTGAGCGTAATCTACC |
| *Sp7* | GACTACCCACCCTTCCCTCACTC | TAGACACTAGGCAGGCAGTCAGAC |
| *Runx2* | CCTTCAAGGTTGTAGCCCTC | GGAGTAGTTCTCATCATTCCCG |
| *Col1a1* | CCCGGGGGATCCATGAGAGATTCTGTTACAGACG | CGGTGGCGGCCGTCAGCAGATCAGGATCAGC |
| *Lnc159948* | AGCGTCCATCCTCACTCTCTTCTC | CTGGCTGTATCGGTTCTCTGGTAAC |
| *Lnc200023* | CACCGTGAGGAAGCAGTCTGAATC | TCTAAGCTCGCACAAGCACAACC |
| *Lnc111822* | GCCAAGGAGGAAAACCGAGGAATC | GGAGCACACTTGTCAGGATGAGAAG |
| *Lnc153455* | GTCTTCCTCCCATCCCAGAGTCAG | TCCTTGCGTGTCAGTGTCTTGC |
| *Lnc127036* | TGAAGAGGACAAGAAGCCAACACAG | GGTTTGGGAGAGCCTTGACTTTCG |
| *Lnc226545* | TCTTCTGTATGCCCGTGGTTTGATG | ACTAATTCTGGTGCCTGTCCTTTGG |
| *Lnc184461* | CAGCCGAGGAAAGAAAGGAGAAGG | AACTGCTCAGCCAAGAACACTAAGG |
| *Lnc125193* | AGGTTGTGCGGAGACTGAGAGG | CTGCGGCTACTGCTCTGTCAAC |
| *Tlr4* | GCCATCATTATGAGTGCCAATT | AGGGATAAGAACGCTGAGAATT |
| *Sphk1* | CTTCTCATTGGACTGTGGTACC | CGTAGAACAGATGCATAACACC |
| *Spi1* | CAACCGCAAGAAGATGACCTACC | ACCTCGCCGCTGAACTGG |
| *Foxa2* | ACAGCCACCACCACCATCAG | GCCCGCTTTGTTCGTGACTG |
| *Gapdh* | AGGTGAAGGTCGGAGTCAAC | CGCTCCTGGAAGATGGTGAT |
| *U6* | GGAACGATACAGAGAAGATTAGC | TGGAACGCTTCACGAATTTGCG |

**Appendix Table S2 Antibodies of proteins used in western blot and immunofluorescence analyses**

| Antibodies | Dilution | Source | Indentifier |
| --- | --- | --- | --- |
| Anti-β-catenin antibody | 1:1000 | Huabio | Cat# ET1601-5 |
| Anti-ALP antibody | 1:500 | Huabio | Cat# ET1601-21 |
| Anti-Osterix antibody | 1:1000 | Huabio | Cat# ER1914-47 |
| Anti-Runx2 antibody | 1:500 | Huabio | Cat# ET1612-47 |
| Anti-Col1a1 antibody | 1:1000 | ZEN-BIOSCIENCE | Cat# 501352 |
| Anti-TLR4 antibody^a^ | 1:1000 | Abcepta | Cat# AP1504a |
| Anti-SPHK1 antibody^a^ | 1:700 | Huabio | Cat# ET1704-76 |
| Anti-PU.1 antibody^a^ | 1:1000 | Abcam | Cat# ab227835 |
| Anti-GAPDH antibody | 1:10000 | Huabio | Cat# EM1101 |

^a^ indicated that the antibody was used both in western blot and immunofluorescence analyses.

**Appendix Table S3. Sequences of primers used in the ChIP-PCR assays**

| Gene | Forward primer sequence (5′–3′) | Reverse primer sequence (5′–3′) |
| --- | --- | --- |
| *Gapdh* | CTGCTGAAGTGCTCCCTACC | CCCTTTTCTGCCTTCCTACC |
| *Tlr4 (1)* | CCCCGTGAACGAATGAACAC | TCTCCCAGAGCACAGTATTAGG |
| *Tlr4 (2)* | TTCCTTCCCTTTCCAAATCAAG | ATTCCCTGCCTCTAACTTGTG |
| *Tlr4 (3)* | AGAACTGAGCAAACAAGGAAGG | TCCATCTCTGCCAACATAACTG |


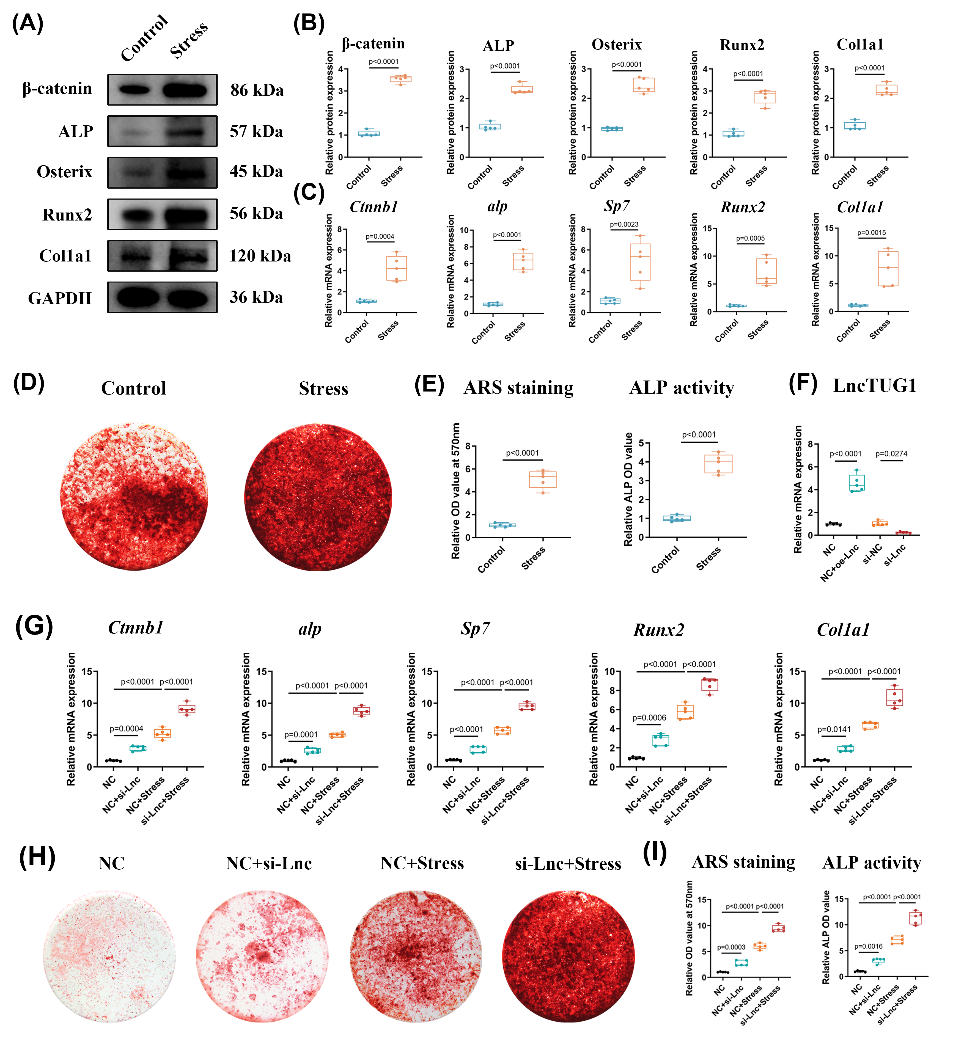


**Appendix Figure 1** Favorable compressive force promotes the mineralization of IDG-CM6 cells and knocking down LncTUG1 further enhances such effects. (A, B) The expressions of β-catenin, ALP, Osterix, Runx2 and Col1a1 in IDG-CM6 cells were assessed by western blot analysis using GAPDH as a loading control and the corresponding quantitative analysis was performed. (C) The qRT-PCR analysis was performed to examine the mRNA levels of *Ctnnb1*, *alp*, *Sp7*, *Runx2* and *Col1a1* in IDG-CM6 cells. (D, E) Representative images and quantitative analysis of Alizarin Red S (ARS) staining as well as the activity of ALP were conducted in IDG-CM6 cells. (F) The mRNA level of LncTUG1 in IDG-CM6 cells after overexpressed by lentivirus was assessed by qRT-PCR analysis using GAPDH as a loading control. (G) The expressions of *Ctmnb1*, *alp*, *Sp7*, *Runx2* and *Col1a1* in IDG-CM6 cells were evaluated by qRT-PCR. (H-I) Representative images and quantitative analysis of Alizarin Red S (ARS) staining as well as the activity of ALP were conducted in IDG-CM6 cells after LncTUG1 was knocked down. Statistical comparison was performed using one-way ANOVA with Tukey’s post hoc test. *p* < 0.05 was considered statistically significant. At least five independent experiments were conducted.


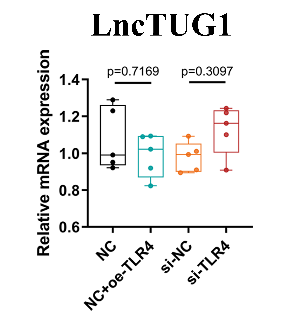


**Appendix Figure 2** The level of LncTUG1 examined by qRT-PCR after overexpression or knockdown of TLR4 expression. Statistical comparison was performed using one-way ANOVA with Tukey’s post hoc test. *p* < 0.05 was considered statistically significant. At least five independent experiments were conducted.


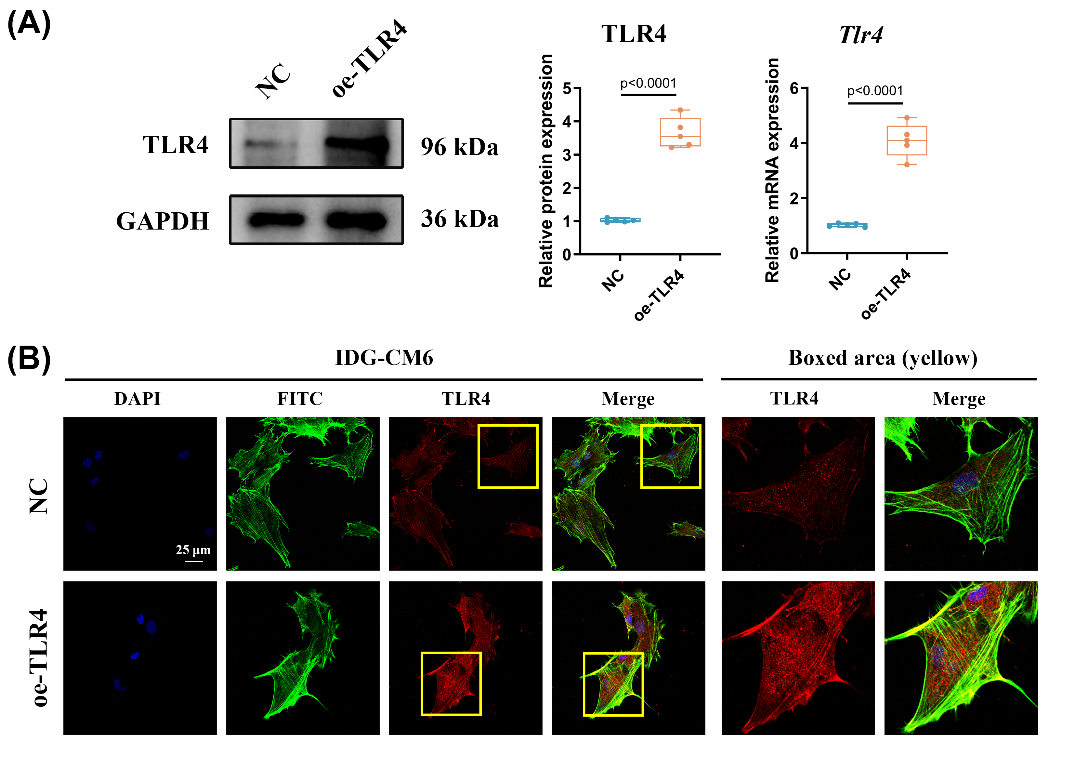


**Appendix Figure 3** (A) The expression of TLR4 in IDG-CM6 cells after overexpressed by specific plasmids was assessed by western blot and qRT-PCR analyses using GAPDH as a loading control and the corresponding quantitative analysis was performed. (B) Representative immunofluorescence images of TLR4 level in IDG-CM6 cells after overexpressed by specific plasmids. Cytoskeleton, green; TLR4, red; nuclei, blue. Scale bar = 25 μm. Statistical comparison was performed using two-tailed Student’s t test with Tukey’s post hoc test. *p* < 0.05 was considered statistically significant. At least five independent experiments were conducted.


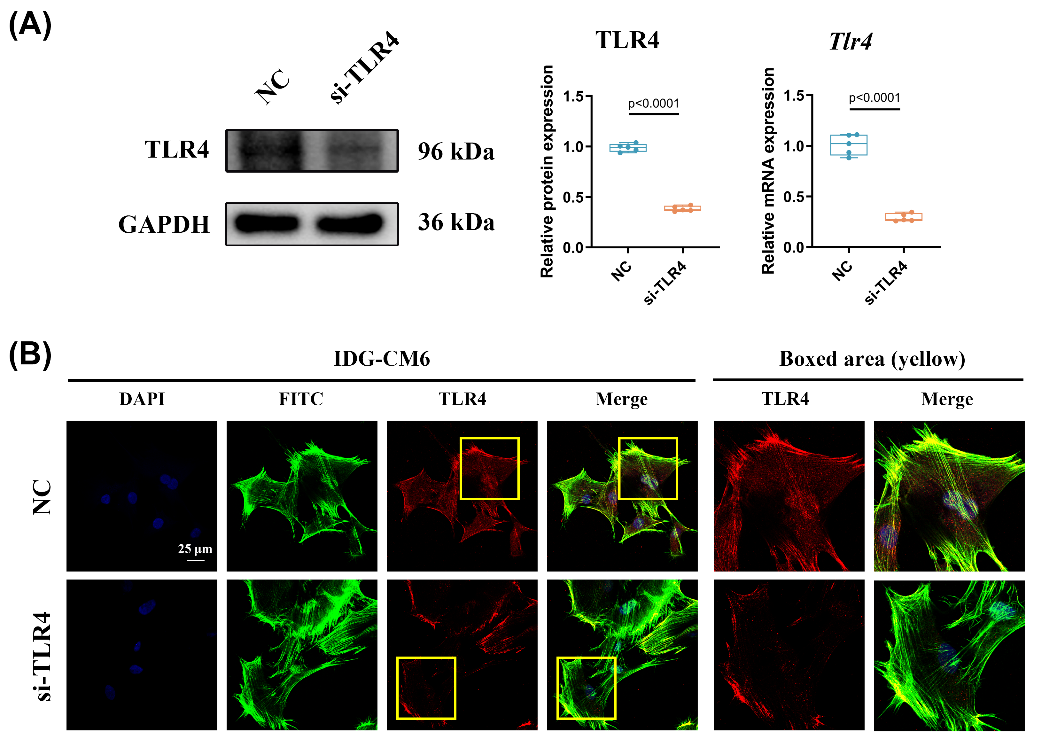


**Appendix Figure 4** (A) The expression of TLR4 in IDG-CM6 cells after knocked down by specific siRNA was assessed by western blot and qRT-PCR analyses using GAPDH as a loading control and the corresponding quantitative analysis was performed. (B) Representative immunofluorescence images of TLR4 level in IDG-CM6 cells after knocked down by specific siRNA. Cytoskeleton, green; TLR4, red; nuclei, blue. Scale bar = 25 μm. Statistical comparison was performed using two-tailed Student’s t test with Tukey’s post hoc test. *p* < 0.05 was considered statistically significant. At least five independent experiments were conducted.


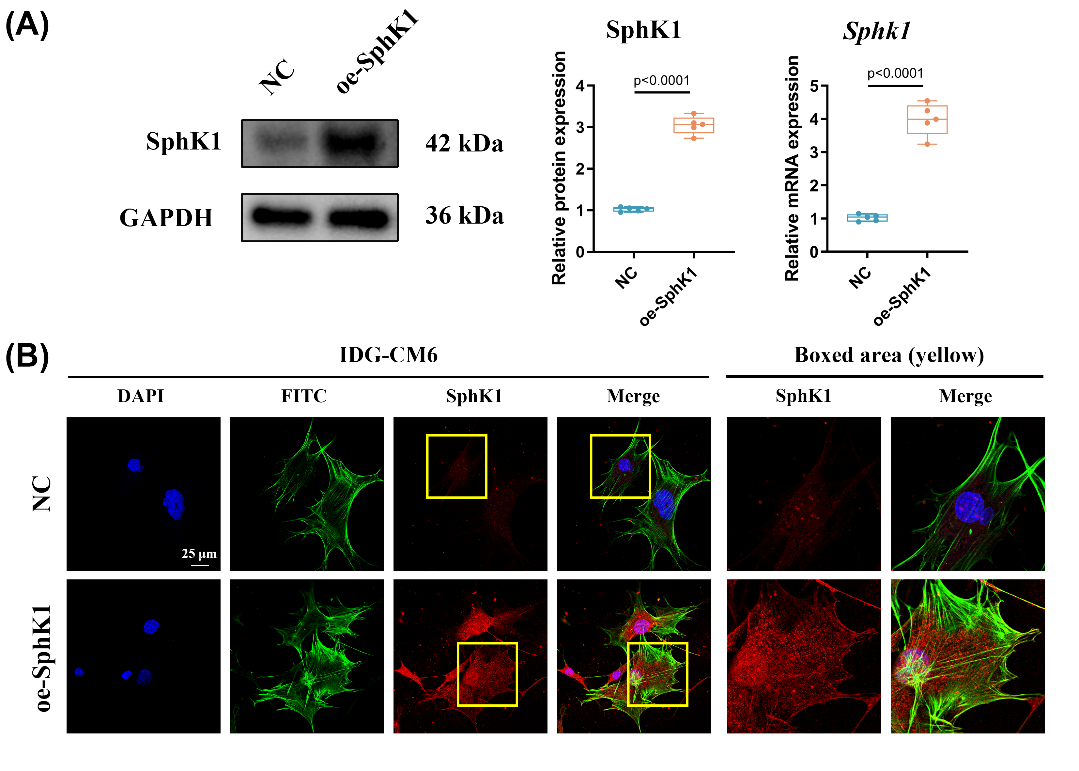


**Appendix Figure 5** (A) The expression of SphK1 in IDG-CM6 cells after overexpressed by specific plasmids was assessed by western blot and qRT-PCR analyses using GAPDH as a loading control and the corresponding quantitative analysis was performed. (B) Representative immunofluorescence images of SphK1 level in IDG-CM6 cells after overexpressed by specific plasmids. Cytoskeleton, green; SphK1, red; nuclei, blue. Scale bar = 25 μm. Statistical comparison was performed using two-tailed Student’s t test with Tukey’s post hoc test. *p* < 0.05 was considered statistically significant. At least five independent experiments were conducted.


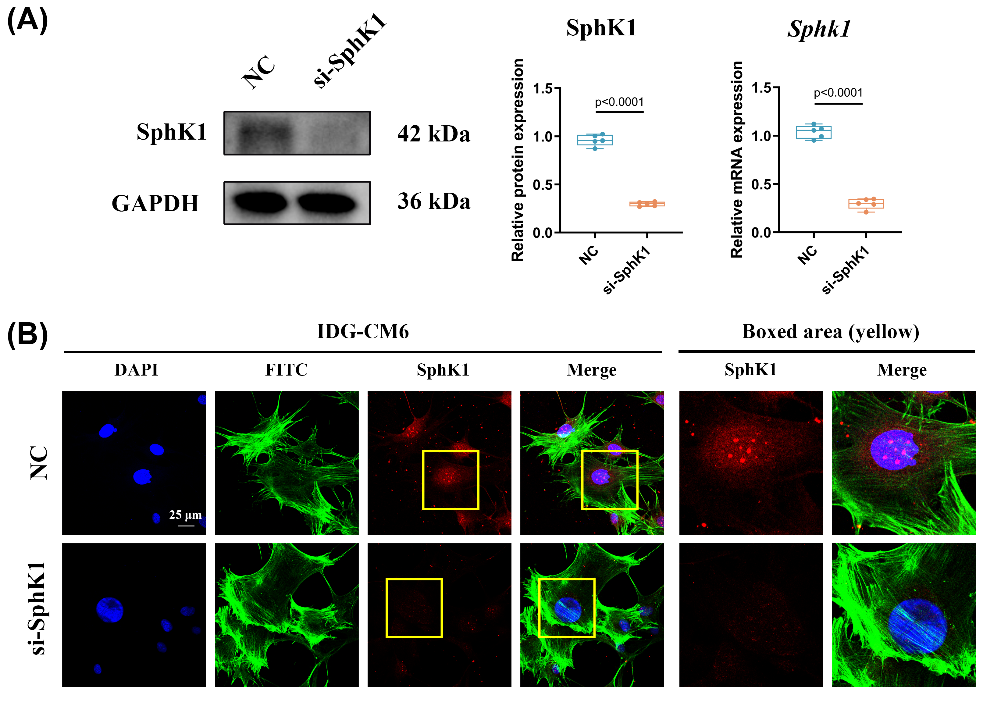


**Appendix Figure 6** (A) The expression of SphK1 in IDG-CM6 cells after knocked down by specific siRNA was assessed by western blot and qRT-PCR analyses using GAPDH as a loading control and the corresponding quantitative analysis was performed. (B) Representative immunofluorescence images of SphK1 level in IDG-CM6 cells after knocked down by specific siRNA. Cytoskeleton, green; SphK1, red; nuclei, blue. Scale bar = 25 μm. Statistical comparison was performed using two-tailed Student’s t test with Tukey’s post hoc test. *p* < 0.05 was considered statistically significant. At least five independent experiments were conducted.


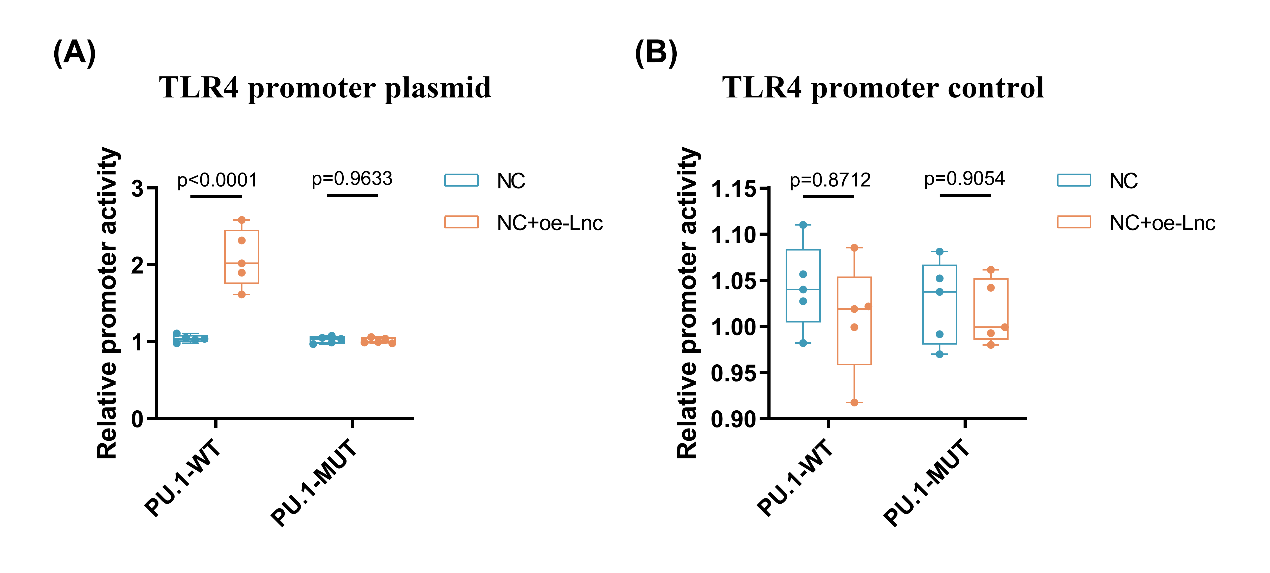


**Appendix Figure 7** TLR4 promoter activity after mutation of binding site of PU.1 with LncTUG1. (A) TLR4 promotor activity in IDG-CM6 cells transfected with TLR4 promotor plasmid detected by luciferase reporter assays. (B) TLR4 promotor activity in IDG-CM6 cells transfected with TLR4 promotor control detected by luciferase reporter assays.
